# Supplementary material for: Potent BRD4 inhibitor suppresses cancer cell-macrophage interaction
Source: Nat Commun. 2020 Apr 14;11:1833. doi: 10.1038/s41467-020-15290-0 (PMC7156724; doi:10.1038/s41467-020-15290-0)
Supplement: Supplementary file 1 — Supplemental Information [file 41467_2020_15290_MOESM1_ESM.pdf]

Supplementary Information

for

Yin et al “Potent BRD4 inhibitor suppresses cancer cell-macrophage interaction”

Inventory

Supplementary Figures 1-16

Supplementary Tables 1-9

Supplementary Methods

Supplementary References

Figure 1 Chemical structures of GSK525762 (I-BET762), CPI-0610, MK8628 (OTX-015) and BMS-986158.
Figure 2 Schematic representation of chemical synthesis of NHWD-870 and NHWD-870-HCl.
Figure 3 Triple negative breast cancer (TNBC), ovarian cancer and small cell lung cancer (SCLC) cells exhibited high BRD4 expression among several human tumor cell lines.
Figure 4 NHWD-870 does not suppress the growth of non-cancerous cells
Figure 5 BRD4 loss inhibited c-MYC expression, and suppressed the growth of A375 melanoma cells.
Figure 6 Metabolic stability of NHWD-870 in (a) mouse, (b) rat, (c) dog, (d) monkey and (e) human liver microsomes.
Figure 7 Pharmacokinetic profile of NHWD-870 using mouse and rat models.
Figure 8 NHWD-870 has tolerable toxicity.
Figure 9 NHWD-870 inhibits growth of SCLC, ovarian tumor, and breast tumor in vivo.
Figure 10 NHWD-870 increased tumor apoptosis and inhibited tumor proliferation.
Figure 11 NHWD-870 treatment decreased the number of TAMs in tumor models.
Figure 12 CSF1 was highly expressed in tumor cells, and CSF1R was highly expressed in TAMs.
Figure 13 NHWD-870 inhibited CSF1/CSF1R signaling pathway to reduce TAM proliferation.
Figure 14 NHWD-870 and BRD4 deletion partially inhibited proliferation of TAMs induced by CSF1.
Figure 15 NHWD-870 inhibited CSF1 expression through suppressing BRD4 and HIF1α in tumor cells.
Figure 16 The levels of phosphorylated BRD4 and CSF1 in tumor cells, as well as the number of TAMs were positively correlated in human ovarian tumors.
Table 1 Metabolic stability of NHWD-870 in mouse, rat, dog, monkey and human liver microsomes.
Table 2 Binding and recovery rates of NHWD-870 in plasma assessed by equilibrium dialysis assays.
Table 3 Summary of pharmacokinetic parameters in mouse and rat after intravenous (IV) and oral (PO) administration of NHWD-870.
Table 4 Mouse body weight changes (%) from Day 0 after NHWD-870 or BMS-986158 treatment.
Table 5 Demographic characteristics of patients with epithelial ovarian cancer and association of CD68 levels with CSF1 and pBRD4 levels and clinical variables of these patients.
Table 6 Univariate analysis of 128 patients with epithelial ovarian cancer.
Table 7 Multivariate analysis with covariate adjustment of 128 patients with epithelial ovarian cancer.
Table 8 List of antibodies
Table 9 List of primers for RT-qPCR
Supplementary Methods:
1. **Synthesis procedure of NHWD-530, NHWD-540, NHWD-560, NHWD-830, NHWD-840, NHWD-850, NHWD-860 and NHWD-870.**
2. **BRD4 binding assay.**
3. **AlamarBlue assay.**
4. **Protein extraction and western blot analysis.**
5. **Colony formation assay.**
6. **MTT and MTS assay.**
7. **Metabolic stability assay.**
8. **Equilibrium dialysis assay.**
9. **In vivo pharmacokinetics study.**
10. **hERG channel assay**.
11. **Plasmids and CRISPR/Cas9-mediated knockout.**
12. **Luciferase reporter assay.**
13. **Patients and tissue samples.**
14. **Immunohistochemical staining.**
15. **ChIP-qPCR analysis.**

Supplementary References

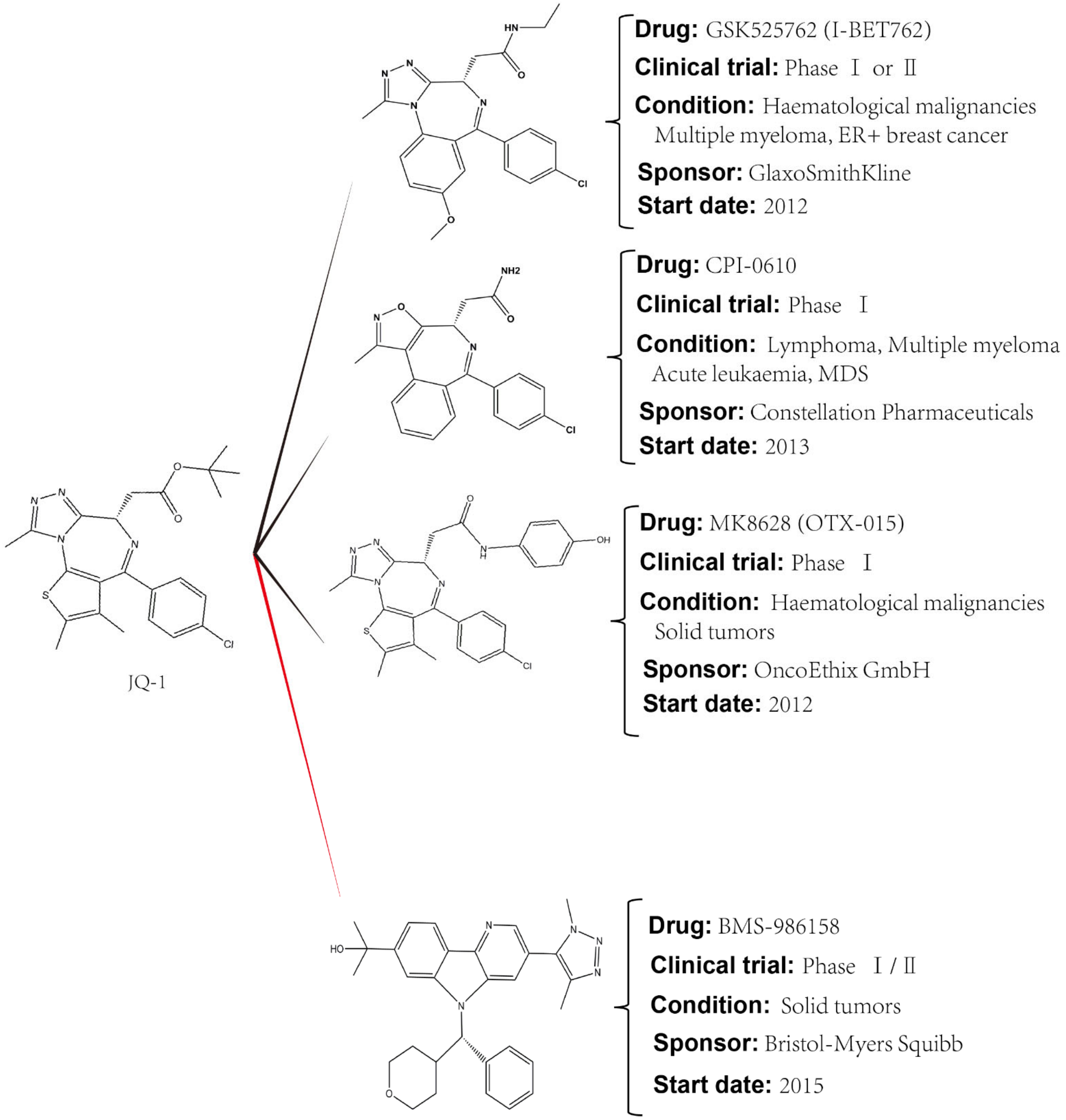

Supplementary Figure 1. Chemical structures of GSK525762 (I-BET762), CPI-0610, MK8628 (OTX-015) and BMS-986158. Their information was obtained from ClinicalTrials.gov, patents and publications. Red line indicated that BMS-986158 adopts distinct chemotype from JQ1.

a

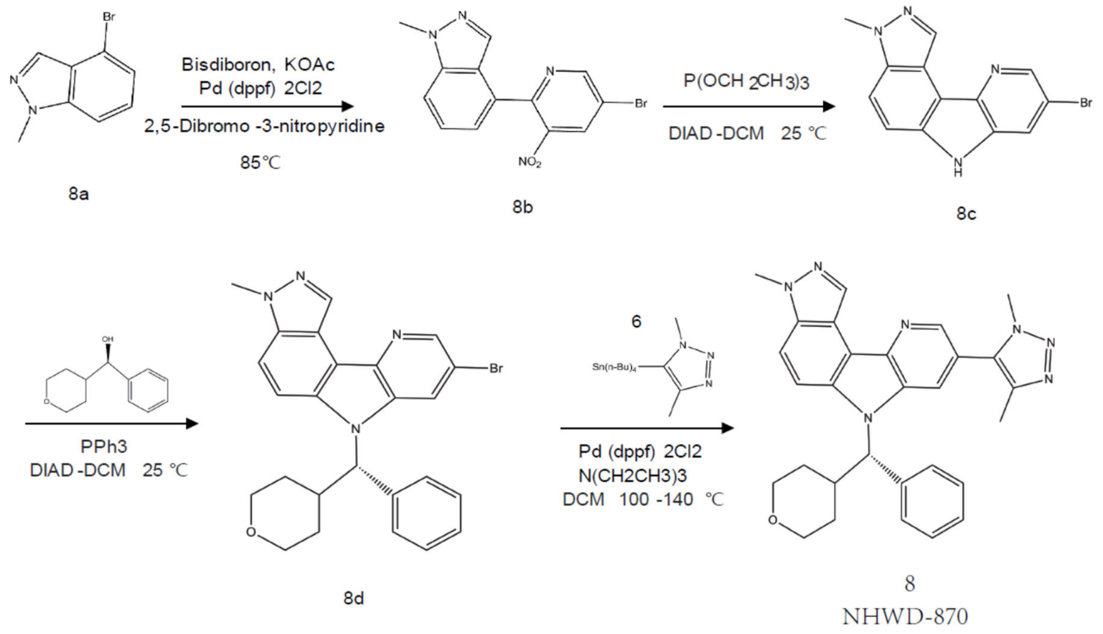

b

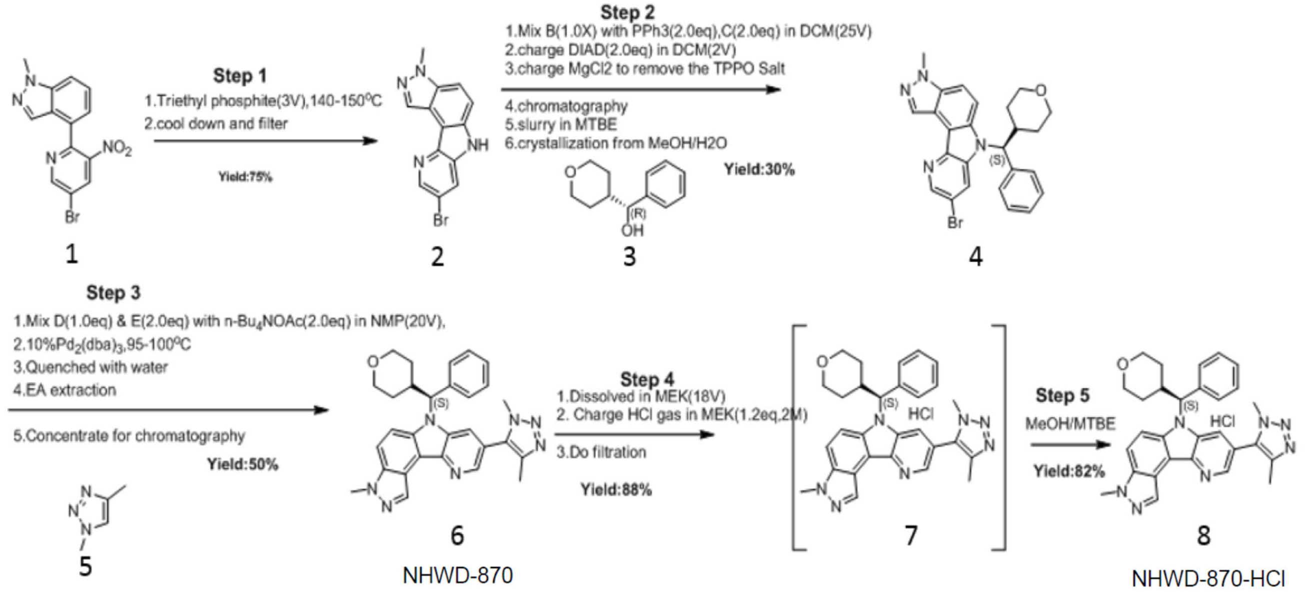

Supplementary Figure 2. Schematic representation of chemical synthesis of NHWD-870 and NHWD-870-HCl. Shown are the original synthesis procedure using Stille coupling (**a**) and the revised synthesis procedure using Suzuki coupling (**b**). The detailed NHWD-870 synthesis procedure is described in **Supplementary Methods**.

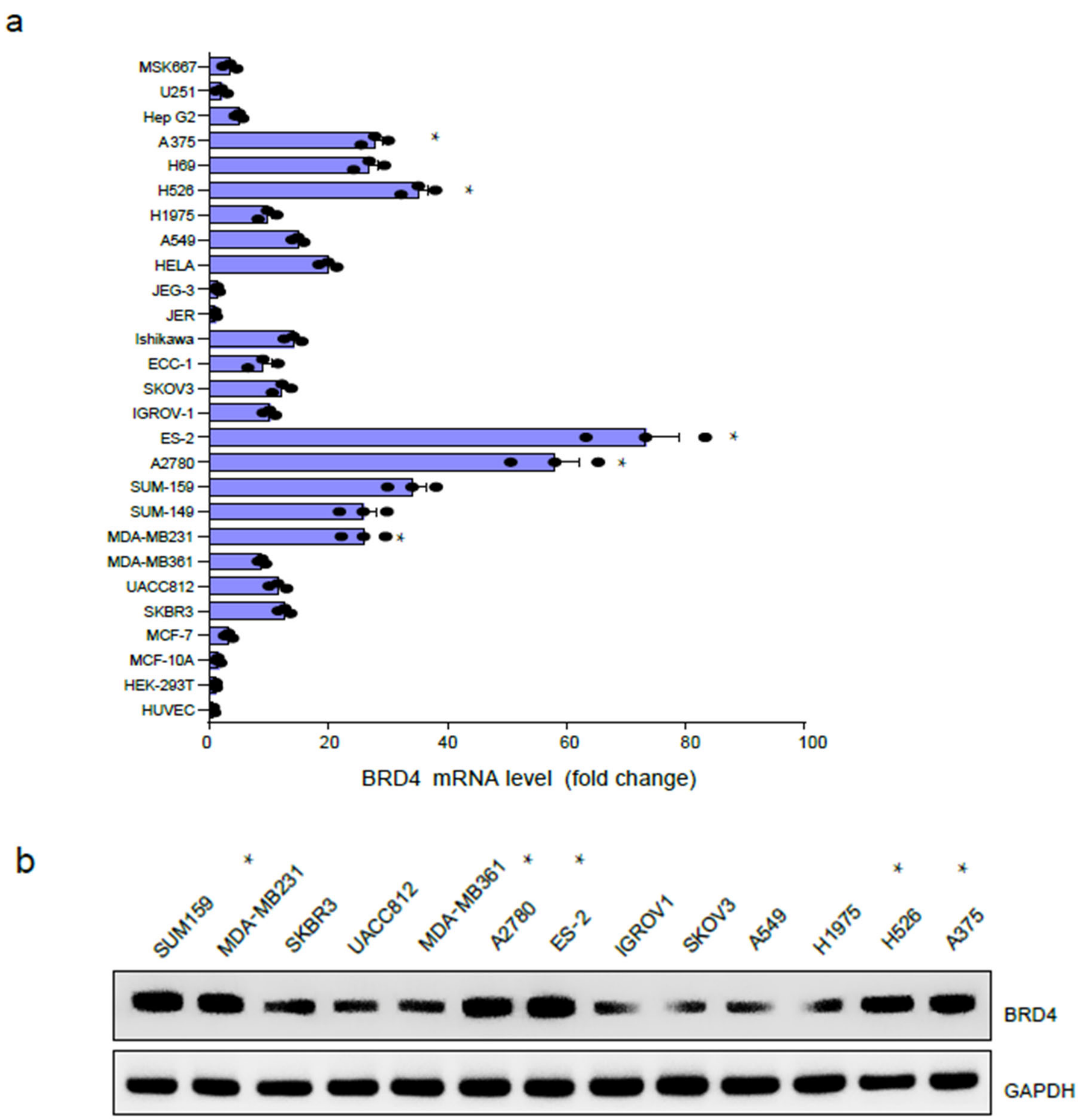

Supplementary Figure 3. Triple negative breast cancer (TNBC), ovarian cancer and small cell lung cancer (SCLC) cells exhibited high BRD4 expression among several human tumor cell lines.

(**a**) RT-qPCR analyses of BRD4 mRNA levels in MCF-7, SKBR3, UACC812, MDA-MB361, MDA-MB231, SUM149, SUM159 breast cancer lines, A2780, ES-2, IGROV1, SKOV3 ovarian carcinoma lines, ECC-1 and Ishikawa endometrial cancer lines, JAR and JEG-3 choriocarcinoma cell lines, Hela cervical cancer cell line, A549 and H1975 non-small cell lung cancer lines, H526 and H69 small cell lung cancer lines, A375 melanoma cell line, HepG2 liver cancer cell line, U-251 glioblastoma cell line, and SH-SY5Y neuroblastoma cell line, human embryonic kidney cell line HEK293T, human umbilical vein endothelial cells (HUVEC) and immortalized human mammary epithelial cell line MCF-10A. The values were normalized to *GAPDH* and then to the relative mRNA level in HEK293T. Data are presented as mean ± SEM of *3* independent experiments.

(**b**) Representative western blot analysis of BRD4 protein levels in SUM159, MDA-MB231, SKBR3, UACC812, MDA-MB361, A2780, ES-2, IGROV1, SKOV3, A549, H1975, H526 and A395 cell lines. GAPDH served as the loading control. * marks the cell lines with high BRD4 expression selected for further analysis. Selected from 3 independent experiments. Source data are provided as a Source Data file.

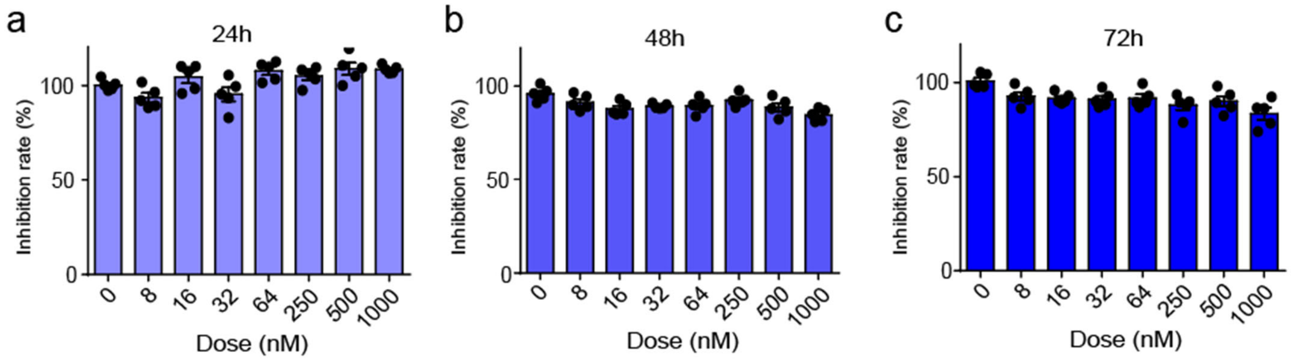

Supplementary Figure 4. NHWD-870 does not suppress the growth of non-cancerous cells. (**a-c**) MTS assays of HaCAT cells (immortalized human keratinocytes) treated with the indicated concentration of NHWD-870 for 24h (**a**), 48h (**b**) or 72h (**c**). Data are presented as mean ± SEM of 5 independent experiments. p values were calculated from two-tailed, unpaired *t* tests. Source data are provided as a Source Data file.

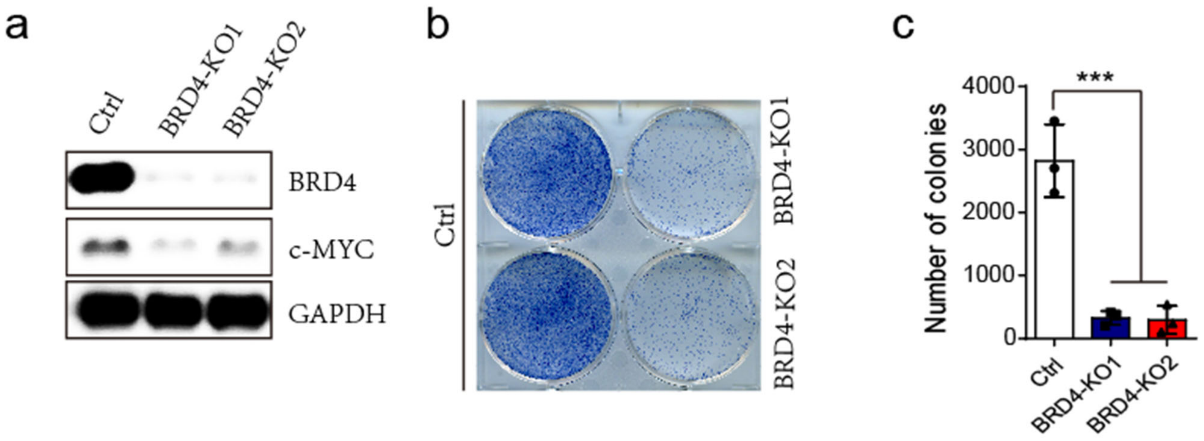

Supplementary Figure 5. BRD4 loss inhibited c-MYC expression, and suppressed the growth of A375 melanoma cells.

(a) Representative western blot analysis of control (Ctrl) or BRD4 knockout (KO1 and KO2) A375 cells with the indicated antibodies. Polyclonal cells with the corresponding sgRNAs were used in these experiments. Selected from 3 independent experiments. **(b,c)** Clonogenic assays of control (Ctrl) or BRD4 knockout (KO1 and KO2) A375 cell. Shown are representative images (**b**) and quantification of colony numbers (**c**). Data are presented as means ± SEM of 3 independent experiments. p values were calculated using two-tailed, unpaired *t* tests. ***, p<0.001. Source data are provided as a Source Data file.

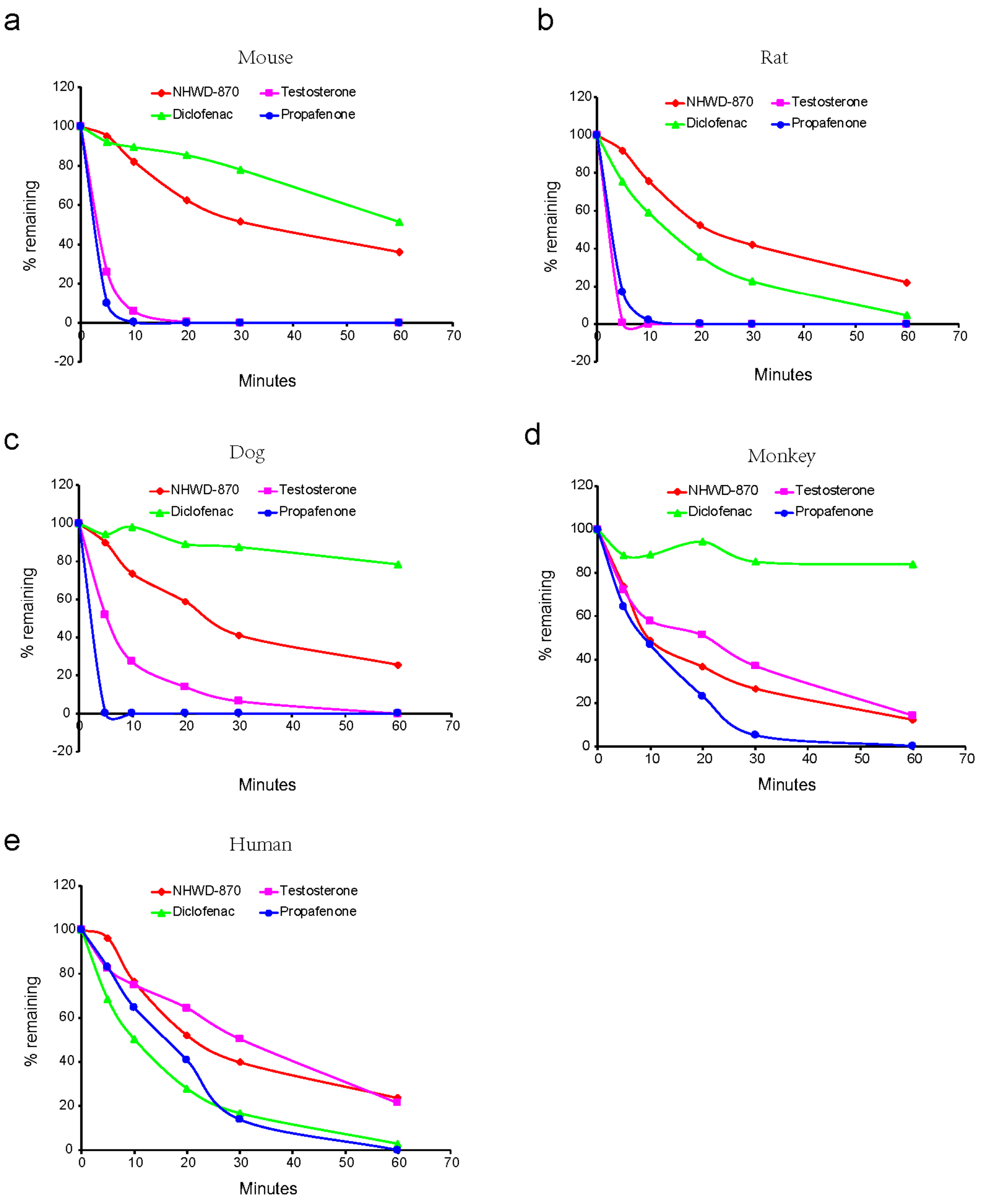

Supplementary Figure 6. Metabolic stability of NHWD-870 in (a) mouse, (b) rat, (c) dog, (d) monkey and (e) human liver microsomes.

Plotted are LC-MS analysis curves of NHWD-870, testosterone, diclofenac and propafenone controls. Quantified parameters are shown in **Supplementary Table 1**. Data are presented as mean ± SEM of 3 independent experiments. Source data are provided as a Source Data file.

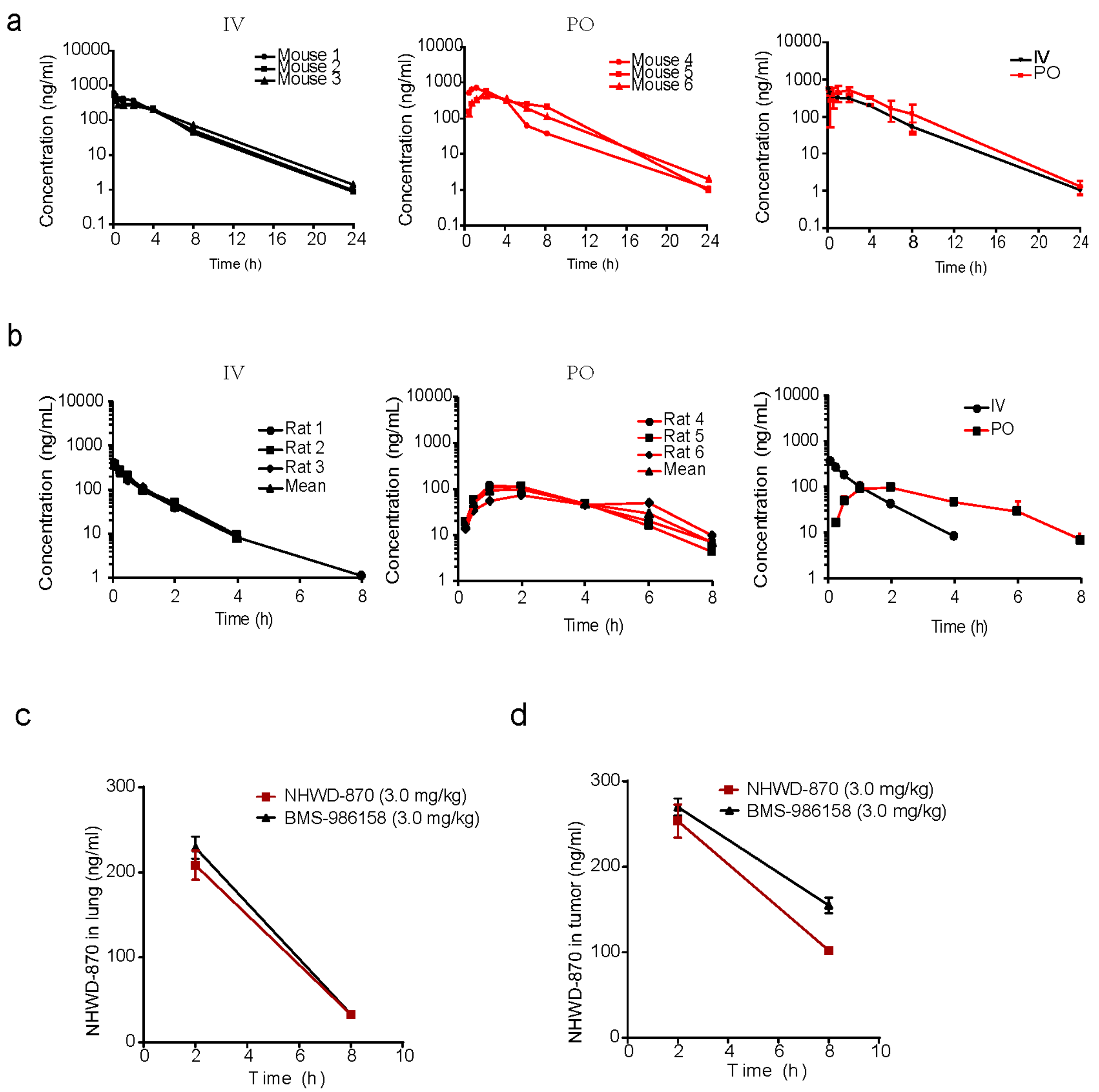

Supplementary Figure 7. Pharmacokinetic profile of NHWD-870 using mouse and rat models. (**a,b**) Plasma concentrations of NHWD-870 in healthy (**a**) mouse and (**b**) rat after intravenous (IV, 1.75 mg/kg for mouse and 1 mg/kg for rat) and oral (PO, 5 mg/kg) administration. Values shown on the right panels are mean ± SEM from 3 different animals. (**c,d**) Mean concentrations of NHWD-870 in the lungs (**c**) and tumors (**d**) of H526 xenograft model at the indicated hours after oral administration of 3 mg/kg NHWD-870 and BMS-98615821 on day 21. These mice were administrated the indicated drug daily for 21 days. Data are represented as mean ± SEM of 3 independent experiments. Source data are provided as a Source Data file.

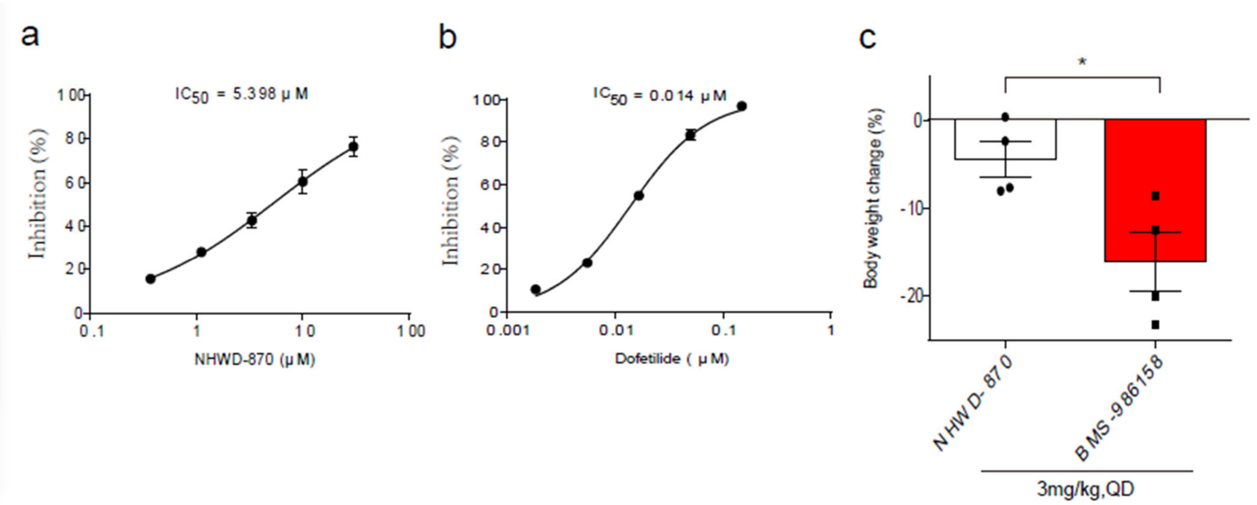

Supplementary Figure 8. NHWD-870 has tolerable toxicity.

(a,b) Effects on hERG channel measured by manual patch-clamp system for NHWD-870 (**a**) and Dofetilide (**b**). Data are presented as mean ±SD from 3 independent experiments. (**c**) Comparison of body weight changes after BMS-986158 or NHWD-870 treatment. H526 SCLC tumor bearing mice were treated with the indicated compounds and body weight changes were calculated based on the body weights of day 0 and day 20 or the last available data point. QD, once daily. Data are presented as mean ± SEM from 4 different mice. p values were calculated using two-tailed, unpaired *t* tests. *, $p<0.05$. Source data are provided as a Source Data file.

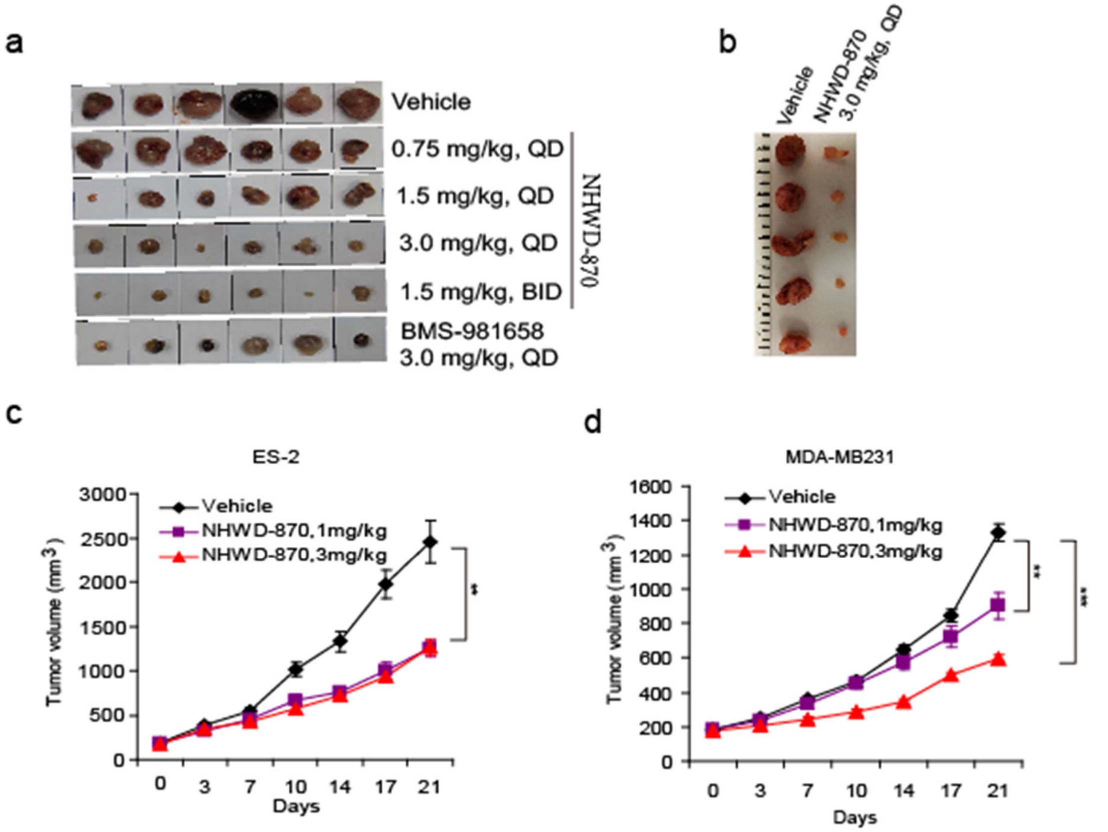

Supplementary Figure 9. NHWD-870 inhibits growth of SCLC, ovarian tumor, and breast tumor in vivo.

(**a,b**) Images of tumors from mice H526 SCLC (**a**) and A2780 ovarian (**b**) tumor bearing mice treated with the indicated compounds for 21 (**a**) or 15 (**b**) days. PO, oral administration; QD, once daily; BID, twice daily; D, day. (**c,d**) Tumor growth curves for ES2 ovarian tumor (**c**), MDA-MB231 breast tumor (**d**) bearing mice treated with the indicated compounds for 21 days. Compounds were orally administrated once daily. Data are presented as mean ± SEM from 6 different mice. p values were calculated using two-tailed, unpaired *t* tests. **, $p<0.01$; ***, $p<0.001$. Source data are provided as a Source Data file.

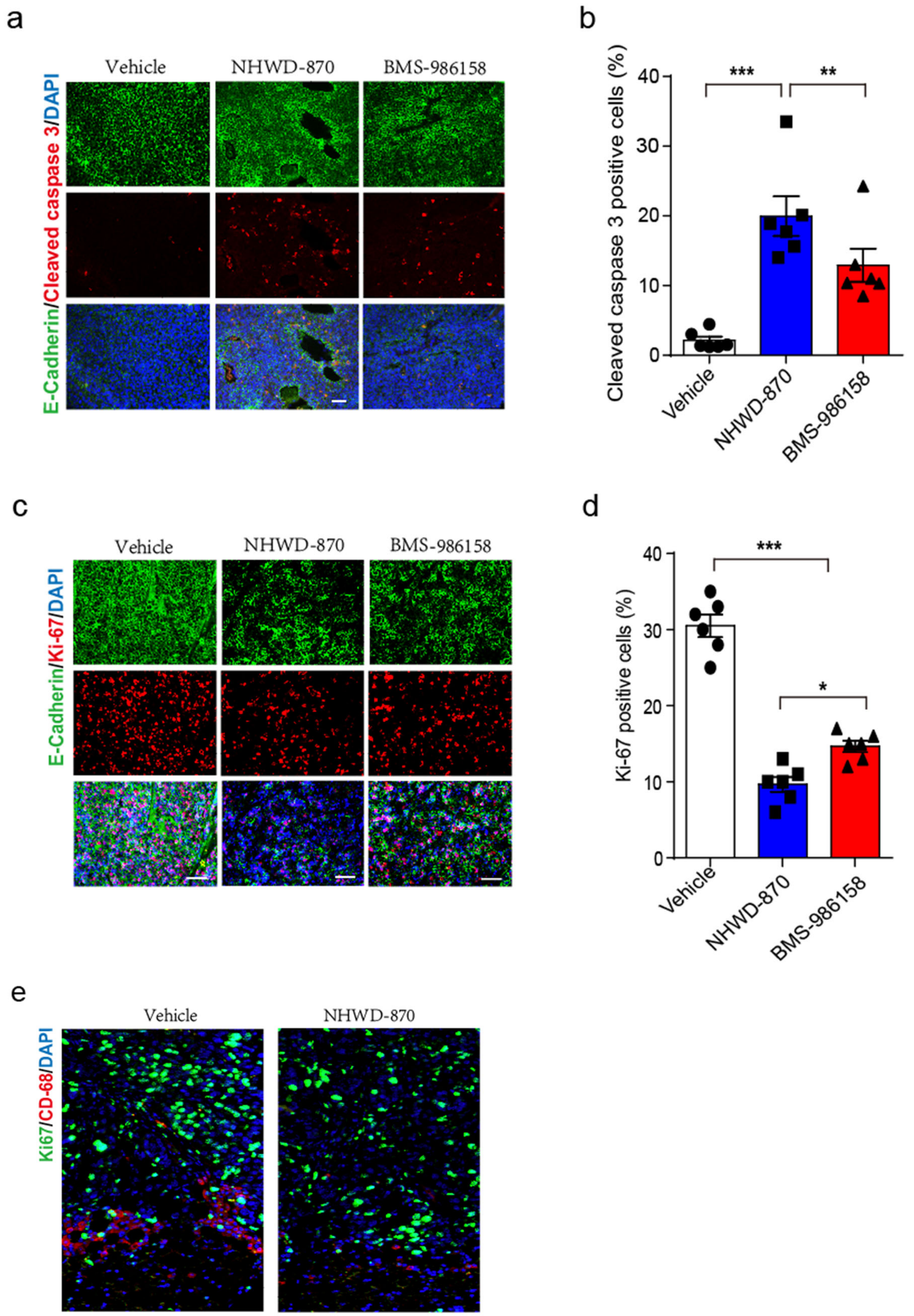

Supplementary Figure 10. NHWD-870 increased tumor apoptosis and inhibited tumor proliferation. (**a**,**b**) Immunofluorescent staining of cleaved caspase 3 (apoptotic cells, red) and E-Cadherin (cancer cells, green) in vehicle, NHWD-870 or BMS-986158 treated H526 tumors from **Figure 4a.** Shown are representative images of cleaved caspase 3 positive cells (**a**) and quantification of cleaved caspase 3 positive cells (**b**). Scale bar is 30 μm. Data are presented as mean ± SEM from 6 independent images. p values were calculated using two-tailed, unpaired *t* tests. **, $p<0.01$; ***, $p<0.001$. (**c**,**d**) Immunofluorescent staining of

Ki67 (proliferating cells, red) and E-Cadherin (cancer cells, green) in vehicle, NHWD-870 or BMS-986158 treated H526 tumors from (Figure **4a**)**.** Shown are representative images of Ki67 positive cells (**c**) and quantification of Ki67 positive cells (**d**). Scale bar is 30 μm. Data are presented as mean ± SEM from 6 independent images. p values were calculated using two-tailed, unpaired *t* tests. *, $p<0.05$; ***, $p<0.001$. (**e**) NHWD-870 treatment reduced TAM proliferation in subcutaneously implanted A2780 tumors. Shown are representative lower power images of **Fig 4c** selected from 5 independent experiments. Source data are provided as a Source Data file.

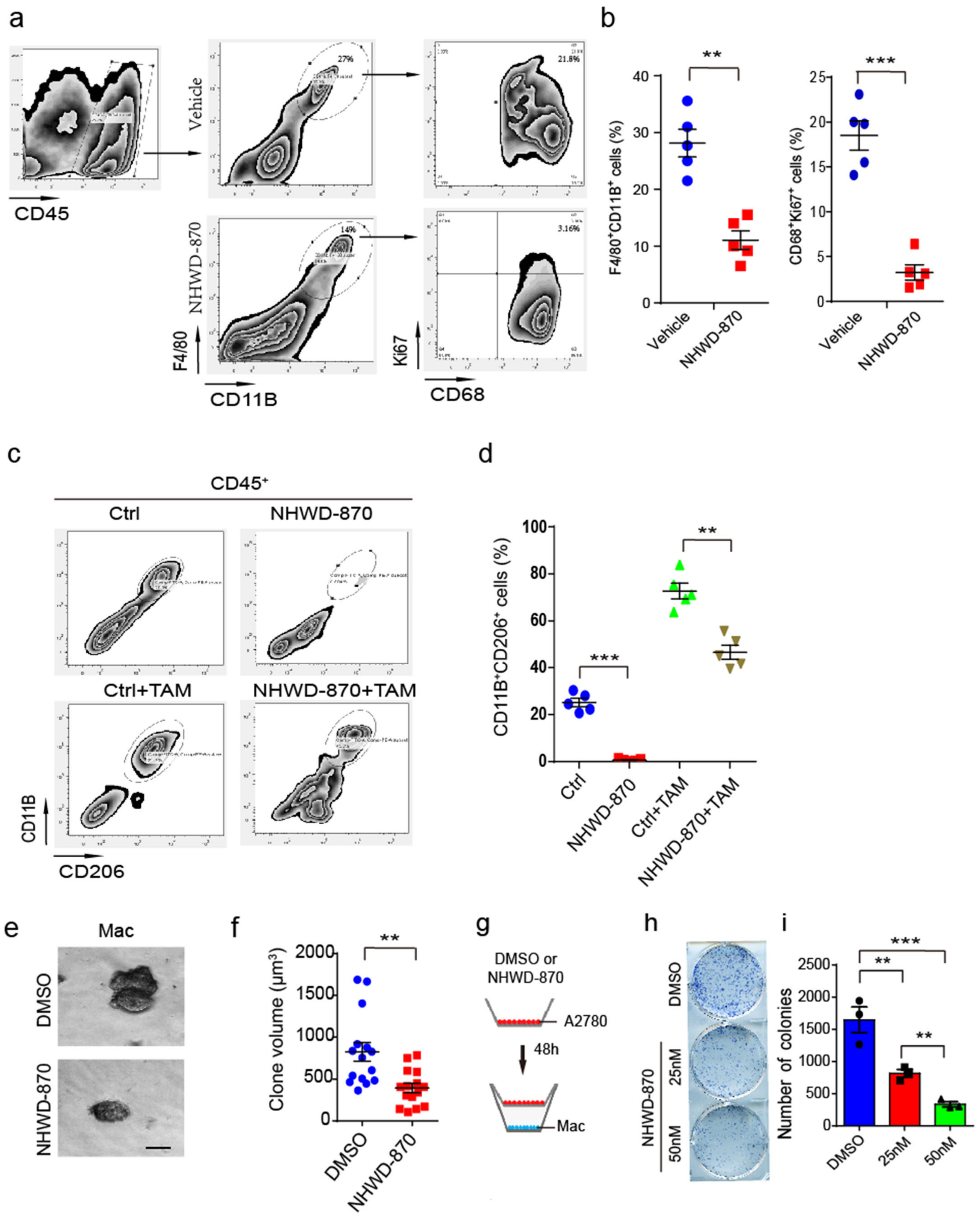

Supplementary Figure 11. NHWD-870 treatment decreased the number of TAMs in tumor models. (**a**,**b**) Tumors from A375 tumor bearing mice in **Figure 3e** were harvested at day 21 and stained with anti-CD45, CD11b, F4/80, CD68 and Ki67 followed by FACS analysis. Shown are representative FACS plots (**a**) and quantification of the percentage of CD11b$^+$F4/80$^+$ and CD11b$^+$F4/80$^+$CD68$^+$Ki67$^+$ cells (**b**). Data are presented as mean ± SEM from 5 different mice. p values were calculated using two-tailed, unpaired *t* tests. **, p<0.01; ***, p<0.001. (**c**,**d**) Tumors from mice described in **Figure 4e** were harvested at day 45 and stained

with anti-CD45, CD11b and CD206 followed by FACS analysis. Shown are representative FACS plots (**c**) and quantification of the percentage of $CD11b^+CD206^+$ cells (**d**). Data are presented as mean ± SEM from 5 different mice. p values were calculated using two-tailed, unpaired *t* tests. **, p<0.01; ***, p<0.001. (**e**-**f**) TAMs (40,000 cells) treated with or without 25 nM NHWD-870 in medium containing 2% Matrigel were seeded onto the 24-well bottom chamber pre-coated with Matrigel. Representative pictures of TAM spheroid (**e**: black/white). Scale bar is 50 μm. Quantification of spheroid volumes (**f**). Data are presented as mean ± SEM from 15 independent experiments. p values were calculated using two-tailed, unpaired *t* tests. **, p<0.01. **(g-i**) NHWD-870 significantly inhibited tumor cell supported TAM proliferation. A2780 cells (pre-treated with DMSO, 25 or 50 nM NHWD-870 for 48h) were seeded into the top chamber (transwell size: 0.4 μm) and TAMs (1×10^5 cells per 6-well) in medium were seeded into the bottom chamber. $CD45^+F4/80^+CD11b^+CD206^+$ TAMs were isolated from tumors of ovarian cancer-bearing donor mice. Shown are schematics of the experiment (**g**), representative images (**h**) and quantification of colonies (**i**). Data are presented as mean ± SEM from 3 independent experiments. p values were calculated using two-tailed, unpaired *t* tests. **, p<0.01; ***, p<0.001. Source data are provided as a Source Data file.

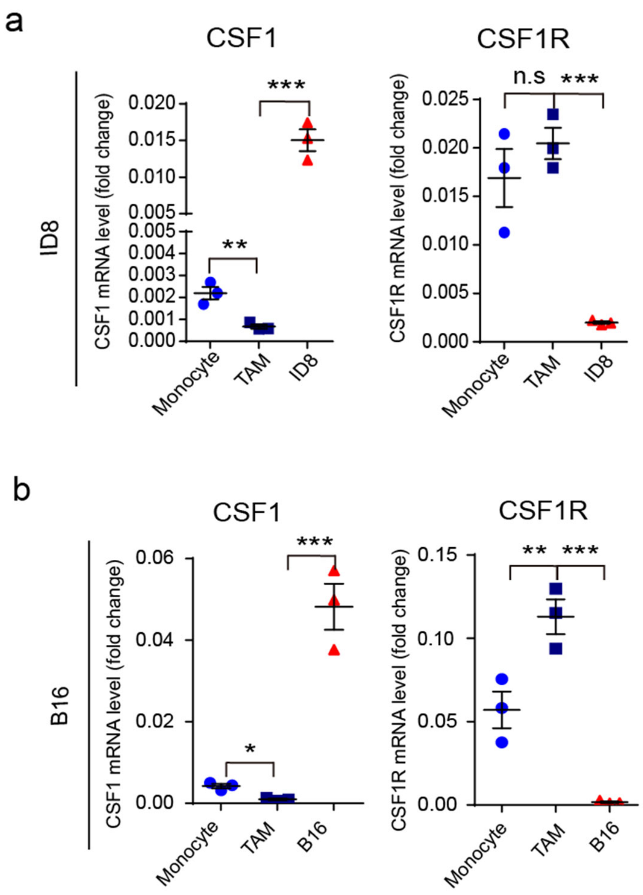

Supplementary Figure 12. CSF1 was highly expressed in tumor cells, and CSF1R was highly expressed in TAMs.

CD11b$^+$ monocytes in the orthotopic OC model were harvested from blood and CD11b$^+$CD206$^+$ macrophages (TAMs) in the orthotopic OC model were isolated from tumors. RT-qPCR analyses of *CSF1* and *CSF1R* in monocytes, TAMs (**a**,**b**), ID8 mouse ovarian cancer cells (**a**), and B16 mouse melanoma cells (**b**). Data are presented as mean ± SEM from 3 independent experiments. p values were calculated using two-tailed, unpaired *t* tests. n.s, not significant; *, p<0.05; **, p<0.01; ***, p<0.001. Source data are provided as a Source Data file.

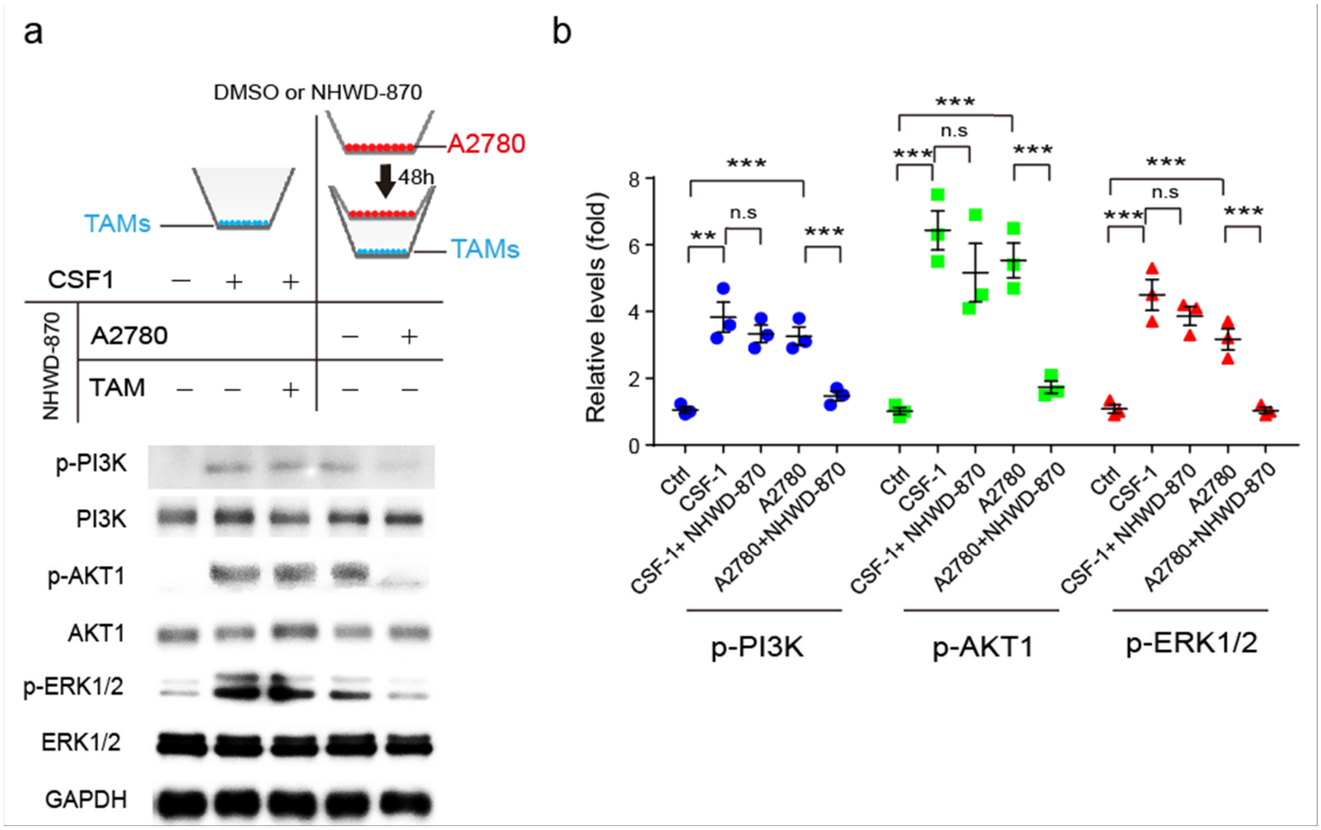

Supplementary Figure 13. NHWD-870 inhibited CSF1/CSF1R signaling pathway to reduce TAM proliferation.

TAMs (2×10^5 cells) (treated with or without 10 ng/ml CSF1 and 100 nM NHWD-870 for 48 h) were seeded into the 6-well bottom chamber, in the presence or absence of A2780 cells (pre-treated with or without 100 nM NHWD-870 for 48 h) seeded into the top chamber (transwell size: 0.4μm). Shown are schematics of the experiment and representative western blot analysis of the indicated proteins (**a**), and quantification of the relative levels of p-PI3K, p-AKT1 and p-ERK over their respective total protein (**b**). Data are presented as mean ± SEM from 3 independent experiments. p values were calculated using two-tailed, unpaired *t* tests. n.s, no significant; **, p<0.01; ***, p<0.001. Source data are provided as a Source Data file.

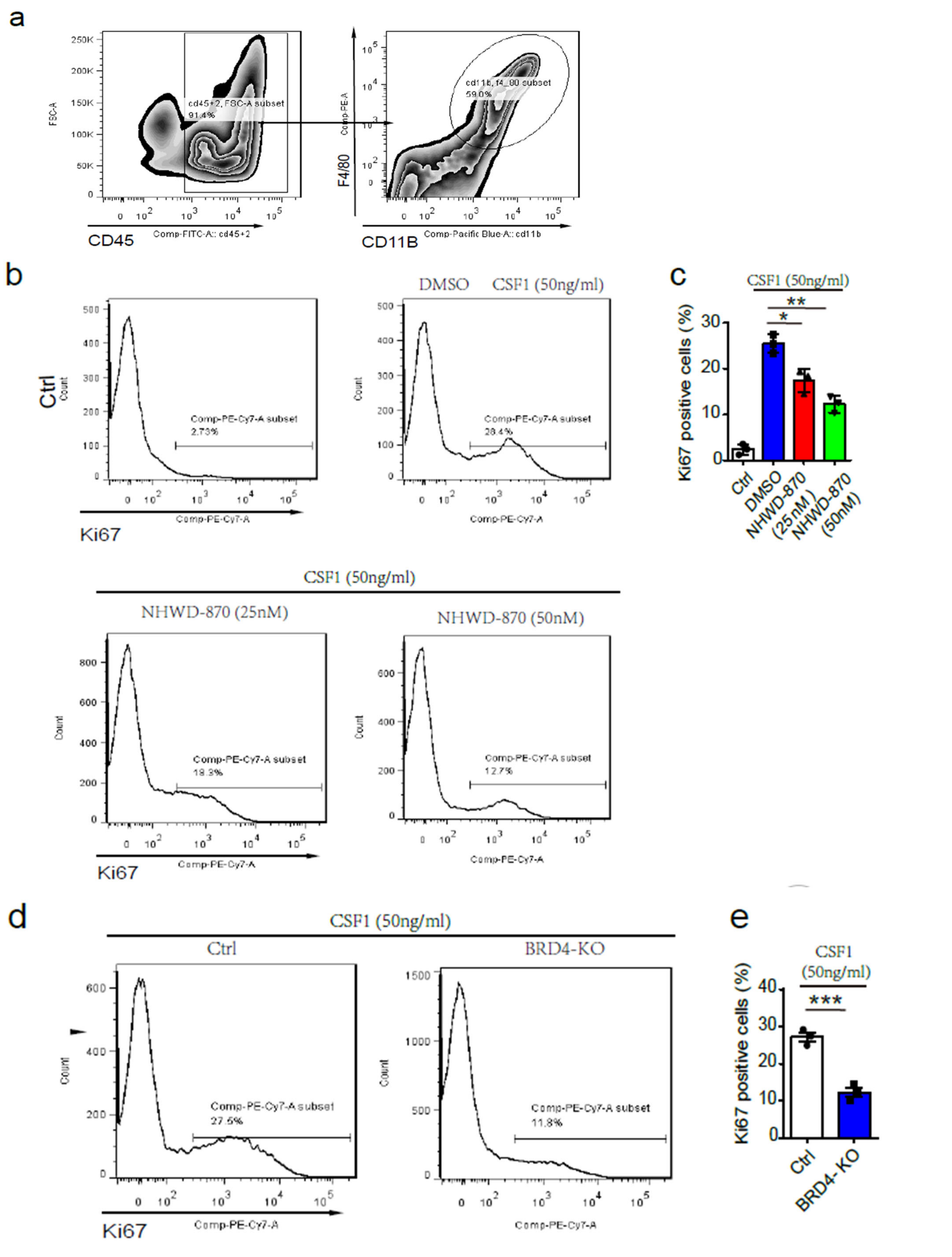

Supplementary Figure 14. NHWD-870 and BRD4 deletion partially inhibited proliferation of TAMs induced by CSF1.

(**a**) CD45$^+$CD11b$^+$F4/80$^+$ TAMs in the orthotopic B16 melanoma model were harvested from tumors. Shown are representative FACS plots of TAMs. (**b**,**c**) Representative FACS plots (**b**) and quantification (**c**) of Ki67$^+$ macrophages treated with 50 ng/ml CSF1, and DMSO or 25 or 50 nM NHWD-870. Data are presented as

mean ± SEM from 3 independent experiments. p values were calculated using two-tailed, unpaired *t* tests. *, $p<0.05$; **, $p<0.01$. (**d,e**) Representative FACS plots (**d**) and quantification (**e**) of $Ki67^{+}$ control (Ctrl) or BRD4 knockout (KO) macrophages treated with 50 ng/ml CSF1. Data are presented as mean ± SEM from 3 independent experiments. p values were calculated using two-tailed, unpaired *t* tests. ***, $p<0.001$. Source data are provided as a Source Data file.

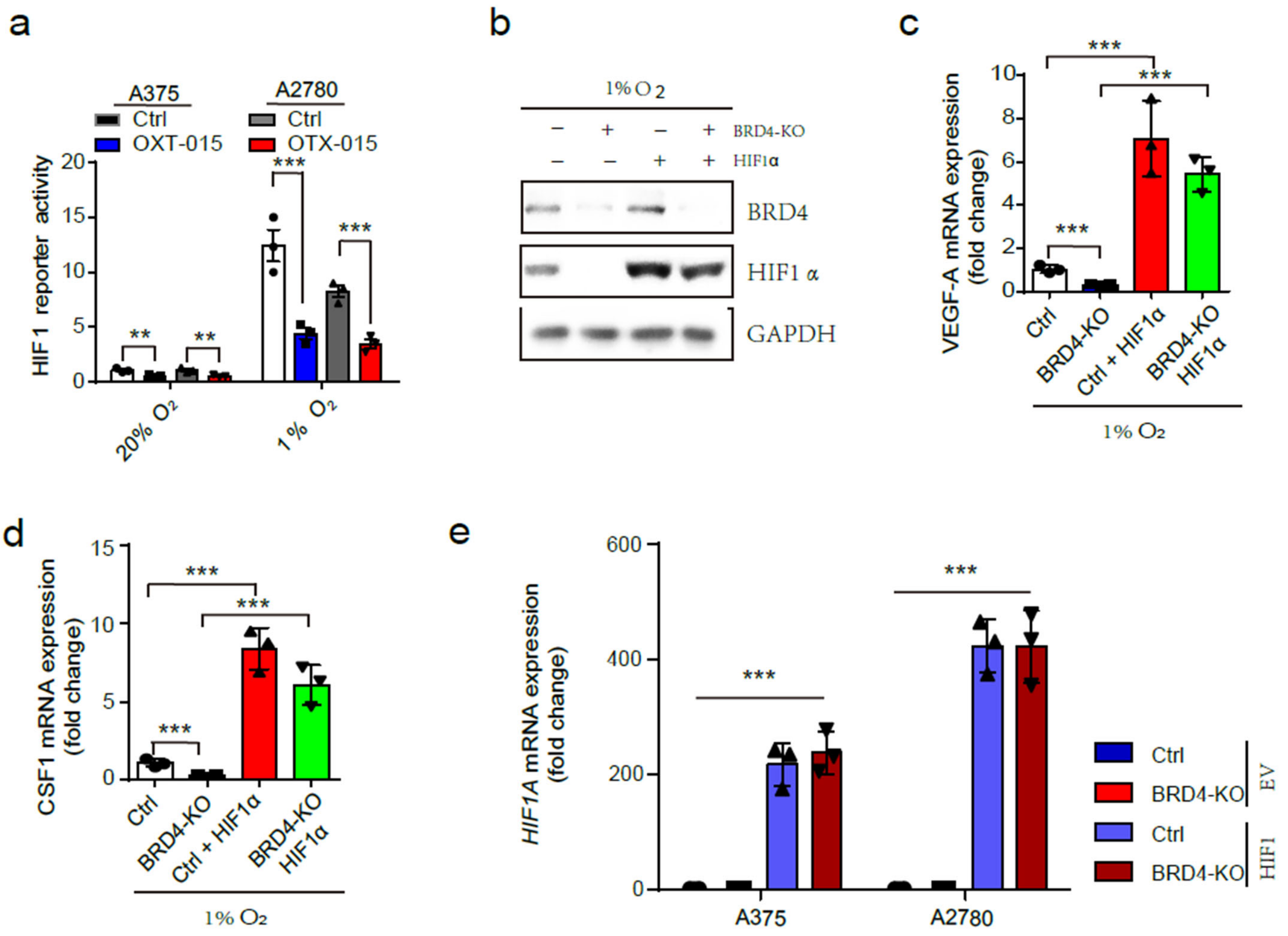

Supplementary Figure 15. NHWD-870 inhibited CSF1 expression through suppressing BRD4 and HIF1α in tumor cells.
(**a**) Relative HIF1 luciferase reporter activity in control (Ctrl) or OTX-015 treated A375 cells exposed to 20% or 1% O_2 for 24 hours. (**b**) Representative western blot analysis of control and BRD4 knockout (KO) HeLa cells transfected with HIF1α plasmids and exposed to 1% O_2 for 24 hours. Selected from 3 independent experiments. (**c**,**d**) RT-qPCR analyses of *VEGFA* (c) and *CSF1* (**d**) in cells shown in panels **c** and **d**. (**e**) RT-qPCR analyses of *HIF1α* in A375 or A2780 cells transfected with HIF1α plasmids. Ctrl, Control. EV, Empty vector. Data in (**a**) and (**c-e**) are presented as mean ± SEM from 3 independent experiments. p values were calculated using two-tailed, unpaired *t* tests. **, $p<0.01$; ***, $p<0.001$. Source data are provided as a Source Data file.

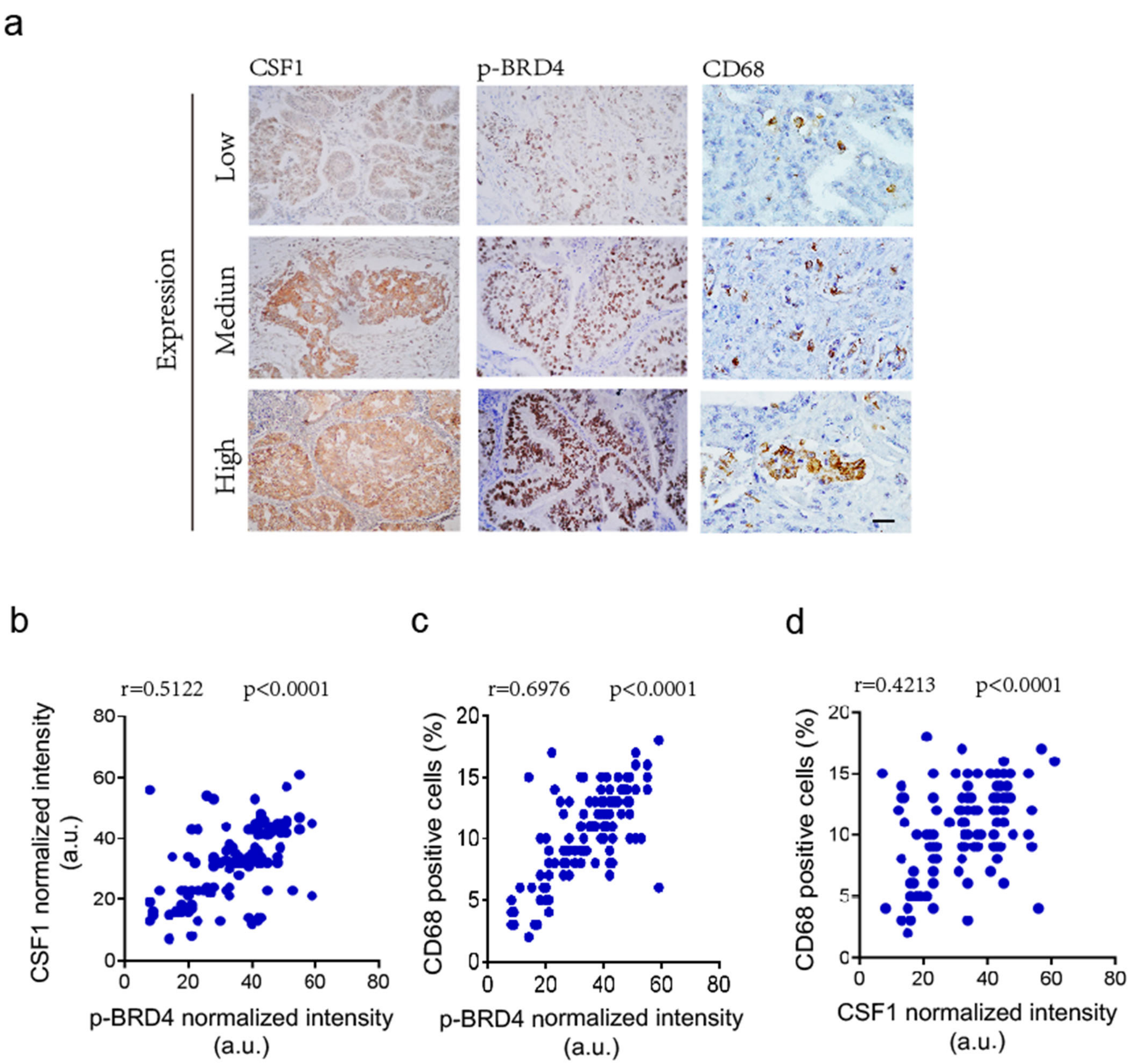

Supplementary Figure 16. The levels of phosphorylated BRD4 and CSF1 in tumor cells, as well as the number of TAMs were positively correlated in human ovarian tumors.

(**a**) Representative immunohistochemical staining of CD68, p-BRD4 and CSF1 in epithelial OC implantation samples with low (left panel), medium (middle panel) and high (right panel) levels of CD68, p-BRD4 and CSF1. Nuclei were stained with hematoxylin. Bar: 50 μm. Selected images from 128 different samples. (**b-d**) Scatter plots showing the relationship between p-BRD4 and CSF1 intensity (**b**), between p-BRD4 intensity and percentage of CD68 positive cells (**c**), between CSF1 intensity and percentage of CD68 positive cells (**d**) in 128 epithelial OC implantation samples. The correlation between CD68 positive cells, CSF1 expression, and p-BRD4 expression was assessed by the odds ratio (p value) using logistic regression with covariate adjustment. Source data are provided as a Source Data file.

Supplementary Table 1 Metabolic stability of NHWD-870 in mouse, rat, dog, monkey and human liver microsomes.

NHWD-870	LM 0.5					
	R^2	$T_{1/2}$ (min)	$CL_{int(mic)}$ (μL/min/mg)	$CL_{int(liver)}$ (mL/min/kg)	Remaining (T=60min)	Remaining (*NCF=60min)
Mouse	0.9635	39.4	35.2	139.2	36.2%	70.1%
Rat	0.9840	27.1	51.1	92.0	22.3%	76.0%
Dog	0.9568	20.8	66.6	95.9	12.4%	84.1%
Monkey	0.9749	30.0	46.2	62.4	25.7%	112.0%
Human	0.9646	27.6	50.3	45.3	23.6%	74.4%

Supplementary Table 2 Binding and recovery rates of NHWD-870 in plasma assessed by equilibrium dialysis assays.

Compound ID	Species/Matrix	%Unbound	SD	% Bound	% Recovery	SD
NHWD-870	Human	2.5	0.1	97.5	92.8	2.7
	CD-1 mouse	2.6	NC	97.4	95.2	NC
	SD rat	4.6	0.2	95.4	92.5	0.6
Warfarin	Human	1.0	0.1	99.0	90.2	3.0
	CD-1 mouse	6.5	0.5	93.5	99.2	3.5
	SD rat	0.5	NC	99.5	86.8	NC

The concentration was calculated using the peak area ratio of analyte and internal standard

The results were calculated by the following equations:

% Unbound = 100 *F_C / T_C, % Bound = 100 * (1-F_C / T_C)

% Recovery = 100 * (F_C + T_C) / T_0

T_C = Total compound concentration as determined by the calculated concentration on the retentate side of the membrane

F_C = Free compound concentration as determined by the calculated concentration on the dialysate side of the membrane

T_0 = Total compound concentration as determined before dialysis

Supplementary Table 3 Summary of pharmacokinetic parameters in mouse and rat after intravenous (IV) and oral (PO) administration of NHWD-870.

	IV (1.75 mg/kg Mouse or 1 mg/kg, Rat)			PO (5 mg/kg, Mouse or Rat)		
	**PK Parameters**	**Mean ± SEM**	**CV (%)**	**PK Parameters**	**Mean ± SEM**	**CV (%)**
**Mouse**	**Cl (mL/min/kg)**	35.0±7.45	42.7	**$T_{1/2}$ (h)**	1.29±0.055	8.85
	**$T_{1/2}$ (h)**	1.73±0.325	37.8	**T_{max} (h)**	1.33±0.29	43.3
	**C_0 (ng/mL)**	630±70.5	22.4	**C_{max} (ng/mL)**	467±74	31.8
	**AUC_{last} (ng.h/mL)**	939±241.5	51.5	**AUC_{last} (ng.h/mL)**	1949±172	17.7
	**AUC_{Inf} (ng.h/mL)**	993±280.5	56.5	**AUC_{Inf} (ng.h/mL)**	2007±165	16.5
	**AUC_{Extrap} (%)**	3.87±2.125	110	**AUC_{Extrap} (%)**	3.04±0.685	45.0
	**MRT_{Inf} (h)**	2.22±0.42	37.9	**MRT_{Inf} (h)**	3.16±0.24	15.2
	**V_{dss} (L/kg)**	4.17±0.42	20.2	**F (%)**	70.8±5.8	16.4
**Rat**	**No. points used for $T_{1/2}$**	ND	--	**No. points used for $T_{1/2}$**	ND	3.0
	**C_0 (ng/mL)**	441±17.9	8.10	**C_{max} (ng/mL)**	101±11.8	23.5
	**$T_{1/2}$ (h)**	0.92±0.0805	17.5	**T_{max} (h)**	1.67±0.288	34.6
	**V_{dss} (L/kg)**	3.27±0.0925	5.67	**$T_{1/2}$ (h)**	1.48±0.155	20.9
	**Cl (mL/min/kg)**	50.4±0.965	3.82	**T_{last} (h)**	8	--
	**T_{last} (h)**	ND	--	**AUC_{last} (ng.h/mL)**	382±12.6	6.08
	**AUC_{last} (ng.h/mL)**	323±5.55	3.44	**AUC_{inf} (ng.h/mL)**	398±9.15	4.60
	**AUC_{inf} (ng.h/mL)**	331±6.45	3.90	**MRT_{last} (h)**	3±0.256	17.1
	**MRT_{last} (h)**	0.98±0.045	9.09	**MRT_{inf} (h)**	3.28±0.33	20.1
	**MRT_{inf} (h)**	1.08±0.021	3.88	**AUC_{Extra} (%)**	3.97±1.15	57.9
	**AUC_{Extra} (%)**	2.21±0.73	66.0	**$AUMC_{Extra}$ (%)**	11.8±2.615	44.2
	**$AUMC_{Extra}$ (%)**	11.5±3.025	52.7	**Bioavailability (%)**	24	--

Note:

1) ND = Not determined (Parameters not determined due to inadequately defined terminal elimination phase);
2) PK parameters were estimated by non-compartmental model using WinNonlin 6.1
3) Bioavailability (%) was calculated using AUC_{inf} with nominal dose.

AUC_{last}: Area under the plasma concentration versus time curve from zero to the last quantifiable concentration

AUC_{inf}: Area under the plasma concentration versus time curve from zero to infinity

AUC_{Extrap}: Extrapolated area under the curve

$AUMC_{Extra}$: Extrapolated area under the first moment curve

C_{max}: Maximum plasma concentration

C_0: Predose plasma concentration

CI: Confidence intervals

CV: Coefficient of variation

F(%): Oral Bioavailability, F=(AUC_{INF}-PO*DoseIV)/(mean AUC_{INF}-IV*DosePO)

MRT_{Inf}: Mean residence time from zero time point to infinity

MRT_{last}: Mean residence time from zero to the last quantifiable concentration
T_{max}: Time to C_{max}
$T_{1/2}$: Terminal half-life
T_{last}: Time of the observed plasma concentration
V_{dss}: Apparent volume of distribution of a drug at steady state

Supplementary Table 4 Mouse body weight changes (%) from Day 0 after NHWD-870 or BMS-986158 treatment.

DAYs	0	3	7	10	14	17	20
BMS-986158 (3mg/kg, QDx21D, n=6)	0.00	-2.33	-2.62	-15.38	-3.28	-5.11	-19.95
	0.00	-1.46	-3.13	-3.30	-5.26	-6.72	-12.40
	0.00	1.06	-0.51	0.08	-0.68	-1.06	-8.56
	0.00	-4.40	-8.04	-4.17	-6.36	-1.00	-1.00
	0.00	1.07	-1.69	1.65	0.13	-0.31	1.16
	0.00	0.98	-7.56	-17.07	-8.04	-23.29	------
NHWD-870 (3mg/kg, QDx21D,n=6)	0.00	-0.08	-0.52	-1.13	-3.67	-0.93	-2.30
	0.00	1.52	0.82	3.04	-1.11	3.90	0.45
	0.00	-2.62	-7.05	-10.82	-16.35	-4.84	-8.20
	0.00	-1.34	-4.19	-3.48	-6.33	-5.58	-7.58
	0.00	1.57	1.94	3.84	0.59	-1.24	-7.97
	0.00	0.13	-3.62	-5.35	-10.57	-10.06	-11.87

(yellow)	**Body weight loss is more than 10%, but less than 15%**
(red)	**Body weight loss is more than 15%**
(grey)	**Dead**

Supplementary Table 5 Demographic characteristics of patients with epithelial ovarian cancer and association of CD68 levels with CSF1 and pBRD4 levels and clinical variables of these patients.

Characteristics	CD68 level		No. of patients(N=128)	*P*
	Low (<11%)	High (≥11%)		
Age (years)				0.270
<50	23	22	45	
≥50	34	49	83	
Serum CA-125 level				0.270 (Fisher)
<35	4	5	9	
≥35	53	66	119	
Ascites				0.754
<100	10	14	24	
≥100	47	57	104	
Lymph node metastasis				0.0027
Absent	47	41	88	
Present	10	30	40	
Histopathological differentiation				<0.0001
Well	26	1	27	
Moderate	29	12	41	
Poor	2	58	60	
Histology type				0.0148
Serous adenocarcinoma	40	55	95	
Mucoid adenocarcinoma	12	3	15	
Endometrioid adenocarcinoma	5	13	18	
CSF1				<0.0001
Low	35	16	51	
High	22	55	77	
p-BRD4				0.0008
Low	24	11	35	
High	33	60	93	
Residual tumor size				0.1040
<1 cm	39	45	84	
1-2 cm	13	15	28	
≥2 cm	5	11	16	
Chemotherapy regimen				0.14
TP	25	35	60	
PAC	32	36	68	

Chi-square test or Fisher's exact test was used to analyze the differences of clinic pathologic characteristics. TP: cisplatin and paclitaxel; PAC: cisplatin, epirubicin and cyclophosphamide.

Supplementary Table 6 Univariate analysis of 128 patients with epithelial ovarian cancer.

Variables	OS		*P*
	5-year (%)	χ^2	
CD68 expression		19.557	<.0001
<11%	64.9		
≥11%	23.9		
CSF1			0.0015
Low	56.89	15.437	
High	43.11		
p-BRD4		17.335	0.0009
Low	59.8		
High	40.2		
Residual tumor size		5.76	0.0100
<1 cm	50.89		
1-2 cm	34.67		
≥2 cm	22.22		
Serum CA-125 level		3.823	0.051
<35	77.7		
≥35	39.5		
Ascites		1.254	0.7625
<100	50		
≥100	45.07		
Lymph node metastasis		14.409	0.0001
Absent	54.6		
Present	15.0		
Histopathological differentiation		10.306	0.0058
Well	59.2		
Moderate	51.2		
Poor	28.3		
Histology type		0.622	0.737
Serous adenocarcinoma	40.2		
Mucoid adenocarcinoma	53.3		
Endometrioid adenocarcinoma	42.1		
Age (years)		0.282	0.595
<50	44.4		
≥50	40.9		
Chemotherapy regimen		38.218	<.0001
TP	73.31		
PAC	21.33		

Kaplan-Meier analysis was used to determine the OS. The log-rank test was used to compare survival outcomes. OS: overall survival time; TP: cisplatin and paclitaxel; PAC: cisplatin, epirubicin and cyclophosphamide.

Supplementary Table 7 Multivariate analysis with covariate adjustment of 128 patients with epithelial ovarian cancer.

Variables	OS		*P*
	HR	*95%CI*	
CD68 expression			
≥11%	2.398	1.032,5.573	0.0420
<11%	1.0		
CSF1			
High	2.053	1.056, 3.258	0.0485
Low	1.0		
p-BRD4			
High	2.177	0.979, 3.866	0.0453
Low	1.0		
Lymph node metastasis			
Present	1.608	0.996, 2.679	0.068
Absent	1.0		
Histopathological differentiation			
Well	0.811	0.349, 1.883	0.636
Moderate	0.647	0.224, 1.87	0.422
Poor	1.0		
Chemotherapy regimen			
TP	1.0		
PAC	3.792	2.086, 6.892	0.0001
Age (years)			
<50	1.0		
≥50	1.131	0.696, 1.83	0.619
Residual tumor size			
<1 cm	1.0		
1-2 cm	0.794	0.317,1.522	0.3826
≥2 cm	3.778	1.597,9.803	0.0037

Multivariate Cox regression (proportional hazard model) was used to identify hazard ratios and evaluate the independent impact of p-BRD4, CSF-1 and CD68 expression on OS. Likelihood Ratio test was used to compare survival outcomes. OS: overall survival time; HR: hazard ratio. TP: cisplatin and paclitaxel; PAC: cisplatin, epirubicin and cyclophosphamide.

Supplementary Table 8 List of antibodies

Antibody name	Company	Host Species	Cat No.	Usage	Dilution
β-Actin	Gene Tex Inc	Rabbit	GTX109639	WB	1:2000
AKT1/2/3	Santa Cruz	Rabbit	sc-8312	WB	1:1500
p-AKT1	Cell signaling	Rabbit	#4060	WB	1:1000
BRD4	abcam	Rabbit	ab128874	WB	1:500
BRD4	Cell signaling	Rabbit	#13440	ChIP	
CD68	abcam	Mouse	ab31630	IF	1:100
CD68(KP1)	Santa Cruz	Mouse	sc-20060	IHC	1:50
Cleaved caspase 3	Cell signaling	Rabbit	#9661	IF	1:100
c-MYC	Santa Cruz	Rabbit	SC-40	WB	1:500
E-Cadherin	abcam	Mouse	ab15148	IF	1:200
EGF	abcam	Rabbit	ab9695	IF	1:50
ERK	Santa Cruz	Rabbit	sc-94	WB	1:1000
p-ERK	Cell signaling	Mouse	#9106	WB	1:1000
GAPDH	Cell signaling	Rabbit	#2118	WB	1:2000
Ki67	Cell signaling	Rabbit	#9129	IF	1:100
CSF1	Santa Cruz	Mouse	sc-365779	IF	1:100
CSF1	abcam	Rabbit	ab52864	WB	1:500
CSF1R/CD115	R&D systems Inc	Mouse	MAB3291-100 (clone 61701)	Neutralization	50ng/ml
PI3K	Santa Cruz	Mouse	sc-1637	WB	1:500
p-PI3K	Cell signaling	Rabbit	#4228	WB	1:500
Mouse anti-Human IgG1	abcam	Mouse	ab1927	Control for neutralization	50ng/ml
HIF1α	Novusbio	Rabbit	Nb100-105	WB	1:500
Rabbit IgG	Beyotime	Rabbit	A7016	ChIP	
**FACS**					
PE-F4/80	Biolegend	Rat	123109	FACS	1:200
APC-F4/80	Biolegend	Rat	100311	FACS	1:200
FITC-CD11b	Biolegend	Rat	101205	FACS	1:200
PE-CD206	Biolegend	Rat	141705	FACS	1:200
FITC-Ki67	Ebioscience	Rat	11-5698-80	FACS	1:500
APC-CD45	Biolegend	Rat	103112	FACS	1:200

Supplementary Table 9 List of primers for RT-qPCR

Primer	Forward Sequence	Reverse Sequence
**Mouse**		
CSF1	GGCTTGGCTTGGGATGATTCT	GAGGGTCTGGCAGGTACTC
CSF1R	TGTCATCGAGCCTAGTGGC	CGGGAGATTCAGGGTCCAAG
GAPDH	AGGTCGGTGTGAACGGATTTG	GGGGTCGTTGATGGCAACA
VEGFA	AAGGAGAGCAGAAGTCCCATGA	CACAGGACGGCTTGAAGATGT
**Human**		
BRD4	GAGCTACCCACAGAAGAAACC	GAGTCGATGCTTGAGTTGTGTT
CSF1	TGGCGAGCAGGAGTATCAC	AGGTCTCCATCTGACTGTCAAT
CSF1R	GGGAATCCCAGTGATAGAGCC	TTGGAAGGTAGCGTTGTTGGT
GAPDH	GGAGCGAGATCCCTCCAAAAT	GGCTGTTGTCATACTTCTCATGG
VEGFA	AGGGCAGAATCATCACGAAGT	AGGGTCTCGATTGGATGGCA
HIF1A	GAACGTCGAAAAGAAAAGTCTCG	CCTTATCAAGATGCGAACTCACA

Supplementary Methods:

1. **Synthesis procedure of NHWD-530, NHWD-540, NHWD-560, NHWD-830, NHWD-840, NHWD-850, NHWD-860 and NHWD-870:**

(1) NHWD-530 (Compound 1)

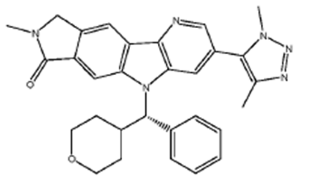

Compound 1

Step A

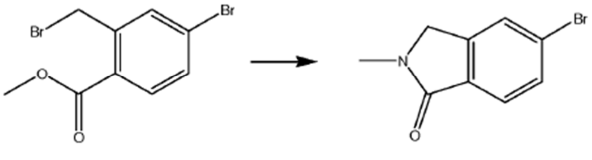

Compound 1a Compound 1b

To a solution of compound 1a (15 g, 50 mmol) in methanol (150 ml) was added methylamine (25 ml, 33% in ethanol), and the reaction was refluxed overnight. After the organic solvent was drained off, the mixture was separated by silica gel column chromatography (PE/EA= 3:1) to provide a yellow oily compound 1b.

HNMR(CDCl3),7.5-7.7(m,3H),4.3(s,2H),3.1(s,3H).MS(ESI)m/z:225.9(M+H)+.

Step B

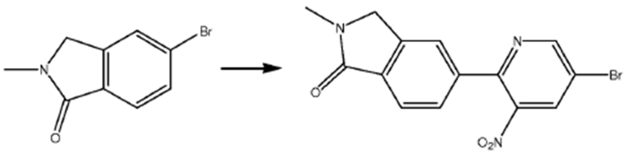

Compound 1b Compound 1c

To a mixed solution of compound 1b (5 g, 22 mmol), bisboronic acid pinacol (8.5 g, 33 mmol) and KOAc (3.2 g, 33 mmol) in 1,4 dioxane (50 ml) was added $Pd(dppf)_2Cl_2$ (1g), and flushed with nitrogen. The reaction flask was sealed and the reaction was stirred overnight at 85°C. After cooling to room temperature, an aqueous Na_2CO_3 solution (2.5M, 10ml), $Pd(dppf)_2Cl_2$ (1g) and 2,5-dibromo-3-nitropyridine (9 g, 33 mmol) were added. After flushed with nitrogen for 10 minutes, the reaction flask was sealed and the mixture was stirred overnight at 85°C. The reaction was poured into water and extracted with ethyl acetate. The organic phase mixture was dried over Na_2SO_4, dried by suction and purified by silica gel column chromatography (PE:EA=10:1 to 1:1) to provide compound 1c as a yellow solid.

HNMR(CDCl3),9.0(s,1H),8.4(s,1H),8.0(d,1H),7.6-7.7(m,2H),4.5(s,2H),3.3(s,3H).MS(ESI)m/z :347.9(M+H)+.

Step C

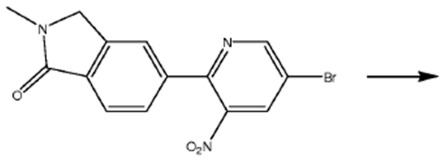

Compound 1c

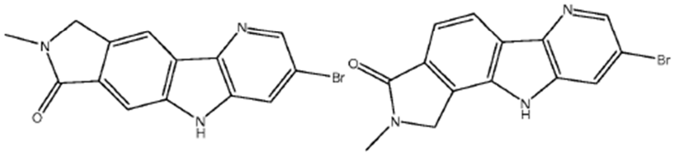

Compound 1d

Compound 1c (3.4 g, 10 mmol) was refluxed in triethylphosphite (50 ml) solution for 3 h. After the solvent was dried by suction, water was added. The solid obtained was washed and dried to provide a compound 1d mixture (of which the isomer ratio was approximately 6:4).

HNMR(DMSO),11.8-12.2(d,1H),8.6(s,1H),8.2-8.4(m,2H),7.4-7.8(m,1H),4.4-4.8(m,2H),3.0-3.4(m,3H).MS(ESI)m/z:316.0 (M+H)+.

Step D

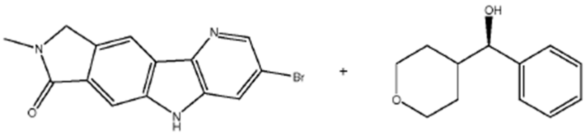

compound 1d mixture intermediate a

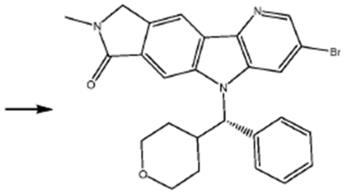

Compound 1e

The preparation of intermediate a referred to the method reported in literature [Orjales, A. et al. J. Med. Chem. 2003, 46, 5512-5532 and WO2015100282]. The compound 1d mixture (1.6 g, 5 mmol) was dissolved in DCM (35 ml), and PPh3 (2 g, 7.5 mmol) and intermediate a (0.7 g, 3.7 mmol) were added. The reaction mixture was stirred in ice-water bath for 1 h, then 10 ml of DIAD (1.5 g, 7.5 mmol) in DCM was slowly added dropwise, and then stirred at room temperature, TLC monitored until the reaction is complete. TLC test showed that only one isomer was consumed. After the solution was drained, the crude was purified by silica gel column chromatography (DCM: MeOH = 50: 1 to 10: 1) to provide white compound 1e.

MS(ESI)m/z:490.0(M+H)+.

Step E

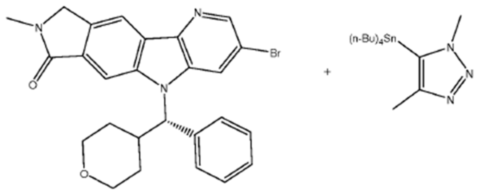

Compound 1e intermediate b

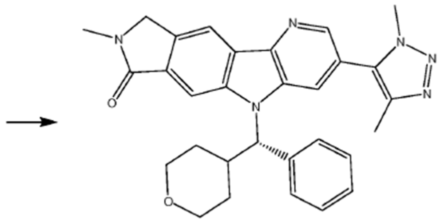

Compound 1

A mixed solution of compound 1e (500 mg, 1.02 mmol), intermediate b (592 mg, 1.5 mmol), triethylamine (0.3 ml, 2 mmol), Pd(dppf)$_2$Cl$_2$ (100 mg) in DMF (10 ml) was flushed with nitrogen. The reaction flask was sealed; the mixture was heated and stirred at 100-140° C for 4 h, poured into water, and extracted with DCM. The combined organic phases were dried and aspirated to dryness to give crude product purified by silica gel column chromatography (DCM:MeOH=50: 1 to 10:1) to provide crude product as a yellow solid and purified by HPLC to obtain compound 1.

HNMR(DMSO),8.4-8.8(m,2H),7.2-7.6(m,7H),5.4-5.7(m,1H),4.6(s,2H),3.8-4.2(m,5H),3.0-3.4(m,5H),2.0-2.4(m,5H),0.8-1.6(m,3H).MS(ESI)m/z:507.2(M+H)+.

(2) NHWD-540 (Compound 2)

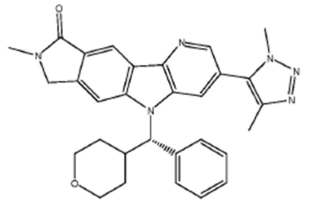

Compound 2

Step A

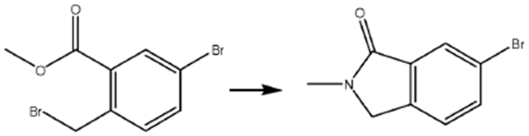

Compound 2a Compound 2b

To a solution of compound 2a (15 g, 50 mmol) in methanol (150 ml) was added methylamine (25 ml, 33% in ethanol), and the reaction was refluxed overnight. After the organic solvent was drained off, the mixture was separated by silica gel column chromatography (PE/EA= 3:1) to provide compound 2b as a yellow oily. MS(ESI) m/z:225.9(M+H)+.

Step B

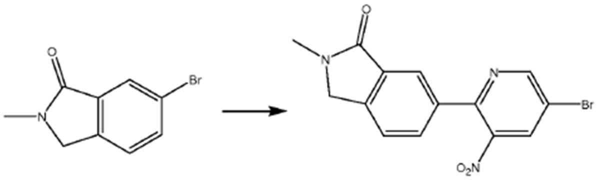

Compound 2b Compound 2c

To a mixed solution of compound 2b (5 g, 22 mmol), bisboronic acid pinacol (8.5 g, 33 mmol) and KOAc (3.2 g, 33 mmol) in 1,4 dioxane (50 ml) was added Pd(dppf)$_2$Cl$_2$ (1g), and flushed with nitrogen. The reaction flask was sealed and the reaction was stirred overnight at 85°C. After cooling to room temperature, an aqueous Na_2CO_3 solution (2.5M, 10ml), Pd(dppf)$_2$Cl$_2$ (1g) and 2,5-dibromo-3-nitropyridine (9 g, 33 mmol) were added. After flushed with nitrogen for 10 minutes, the reaction flask was sealed and the mixture was stirred overnight at 85°C. The reaction was poured into water and extracted with ethyl acetate. The organic phase mixture was dried over Na_2SO_4, dried by suction and purified by silica gel column chromatography (PE:EA=10:1 to 1:1) to provide compound 2c as a yellow solid.

HNMR(DMSO),9.1(s,1H),8.8(s,1H),7.7-7.8(m,3H),4.5(s,2H),3.1(s,3H).MS(ESI)m/z:347.9(M+H)+.

Step C

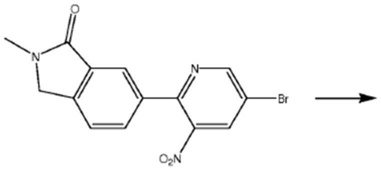

Compound 2c

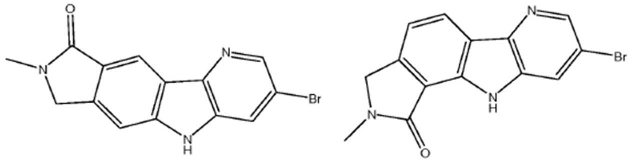

Compound 2d

Compound 2c (3.4 g, 10 mmol) was refluxed in triethylphosphite (50 ml) solution for 3 h. After the solvent was drained, water was added. The solid obtained was washed and dried to provide a compound 2d mixture.

HNMR(DMSO),11.8-12.2(m,1H),8.0-8.6(m,3H),7.2-7.8(m,1H),4.4-4.6(m,2H),3.0-3.1(m,3H). MS(ESI)m/z:316.0 (M+H)+.

Step D

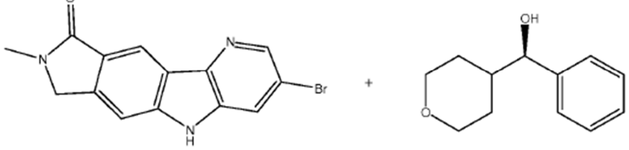

compound 2d mixture intermediate a

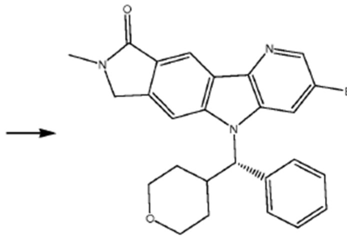

Compound 2e

The compound 2d mixture (1.6 g, 5 mmol) was dissolved in DCM (35 ml), and PPh3 (2 g, 7.5 mmol) and intermediate a (1.4 g, 7.5 mmol) were added. The reaction mixture was stirred in ice-water bath for 1 h, then 10 ml of DIAD (1.5 g, 7.5 mmol) in DCM was slowly added dropwise, and then stirred at room temperature, TLC monitored until the reaction is complete. TLC test showed that only one isomer was consumed. After the solution was drained, the crude was purified by silica gel column chromatography (DCM: MeOH = 50: 1 to 10: 1) to provide white compound 2e.

MS(ESI)m/z:490.0(M+H)+.

Step E

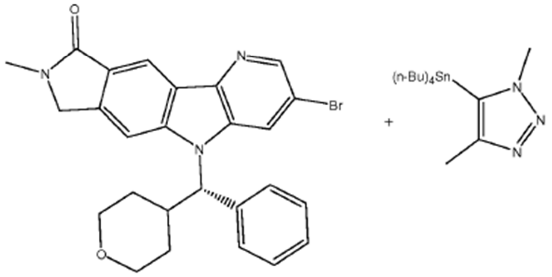

Compound 2e intermediate b

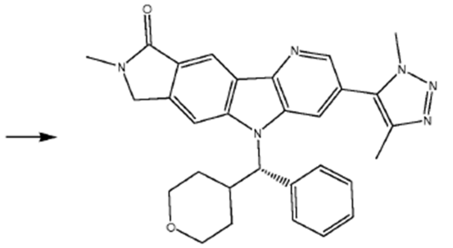

Compound 2

A mixture of compound 2e (500 mg, 1.02 mmol), intermediate b (592 mg, 1.5 mmol), triethylamine (0.3 ml, 2 mmol), and $Pd(dppf)_2Cl_2$ (100 mg) in DMF (10 ml) was flushed with nitrogen. The reaction flask was sealed; the mixture was heated and stirred at 100-140° C for 4 h, poured into water, and extracted with DCM. The combined organic phases were dried and aspirated to dryness to give crude product purified by silica gel column chromatography (DCM:MeOH=50: 1 to 10:1) to provide crude product as a yellow solid and purified by HPLC to obtain compound 2.

HNMR(DMSO),8.9(s,1H),8.6(s,1H),7.7(d,2H),7.2-7.5(m,5H),5.6(m,1H),4.6(s,2H),3.8-4.2(m,5 H),3.0-3.6(m,5H),2.0-2.4(m,5H),1.0-1.6(m,3H).MS(ESI)m/z:507.2(M+H)+.

(3) NHWD-560 (Compound 3)

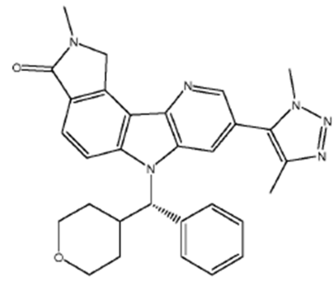

Compound 3

Step A

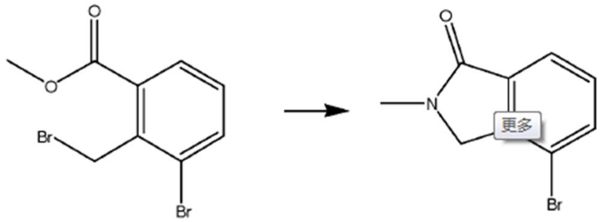

Compound 3a Compound 3b

To a solution of compound 3a (15 g, 50 mmol) in methanol (150 ml) was added methylamine (25 ml, 33% in ethanol), and the reaction was refluxed overnight. After the organic solvent was drained off, the mixture was separated by silica gel column chromatography (PE/EA= 3:1) to provide compound 3b as a yellow oily.

HNMR(DMSO)7.7(m,1H),7.6(m,2H),4.4(s,2H),3.0(s,3H).MS(ESI)m/z:225.9(M+H)+.

Step B

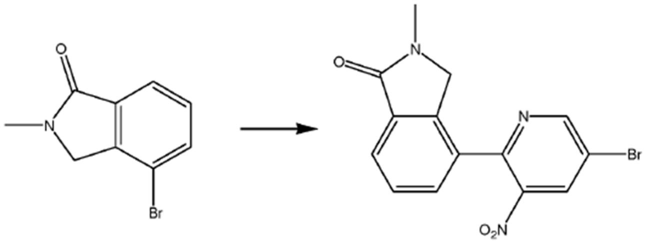

Compound 3b Compound 3c

To a mixed solution of compound 3b (5 g, 22 mmol), bisboronic acid pinacol (8.5 g, 33 mmol) and KOAc (3.2 g, 33 mmol) in 1,4-dioxane (50 ml) was added Pd(dppf)$_2$Cl$_2$ (1g), and flushed with nitrogen. The reaction flask was sealed and the reaction was stirred overnight at 85°C. After cooled to room temperature, an aqueous Na_2CO_3 solution (2.5M, 10ml), Pd(dppf)$_2$Cl$_2$ (1g) and 2,5-dibromo-3-nitropyridine (9 g, 33 mmol) were added. After flushed with nitrogen for 10 minutes, the reaction flask was sealed and the mixture was stirred overnight at 85°C. The reaction was poured into water and extracted with ethyl acetate. The organic phase mixture was dried over Na_2SO_4, dried by suction and purified by silica gel column chromatography (PE:EA=10:2 to 1:1) to provide compound 3c as a yellow solid. MS(ESI)m/z:347.9(M+H)+.

Step C

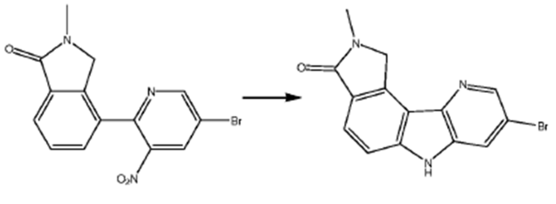

Compound 3c (3.4 g, 10 mmol) was refluxed in triethylphosphite (50 ml) solution for 3 h. After the solvent was drained, water was added. The solid obtained was washed and dried to provide a compound 3d.

HNMR(DMSO),11.8(s,1H),8.5(s,1H),8.1(s,1H),7.7(s,1H),7.6(s,1H),4.7(s,2H),3.1(s,3H).MS(ESI)m/z:316.0 (M+H)+.

Step D

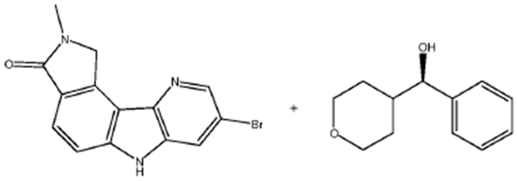

compound 3d intermediate a

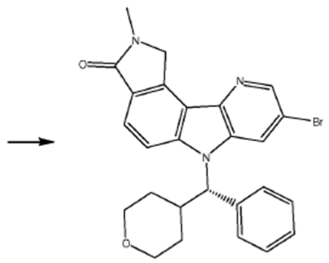

Compound 3e

The compound 3d (1.6 g, 5 mmol) was dissolved in DCM (35 ml), and PPh3 (2 g, 7.5 mmol) and intermediate a (0.7g, 3.7 mmol) were added. The reaction mixture was stirred in ice-water bath for 1 h, then a 10 ml solution of DIAD (1.5 g, 7.5 mmol) in DCM was slowly added dropwise, and then stirred at room temperature, TLC monitored until the reaction is completed. After the solution was dried by suction, the crude was purified by silica gel column chromatography (DCM: MeOH = 50: 1 to 10: 1) to provide white compound 3e. MS(ESI)m/z: 490.0(M+H)+.

Step E

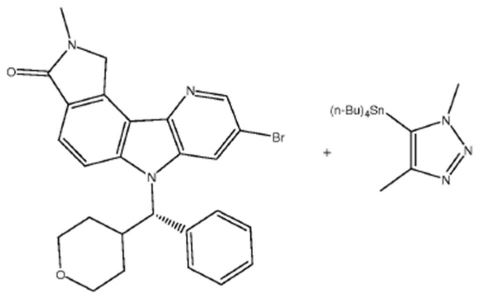

Compound 3e intermediate b

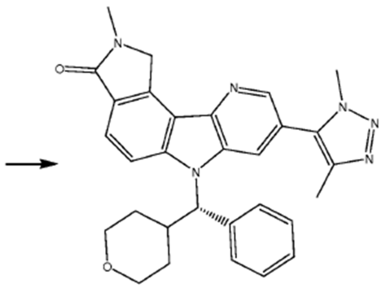

Compound 3

A mixture of compound 3e (500 mg, 1.02 mmol), intermediate b (592 mg, 1.5 mmol), triethylamine (0.3 ml, 2 mmol), Pd(dppf)$_2$Cl$_2$ (100 mg) in DMF (10 ml) was flushed with nitrogen. The reaction flask was sealed, the mixture was heated and stirred at 100-140° C for 4 h, poured into water, and extracted with DCM. The combined organic phases were dried and aspirated to dryness to give crude product purified by silica gel column chromatography (DCM:MeOH=50: 1 to 10:1) to provide crude product as a yellow solid purified by HPLC to obtain compound 3.

HNMR(DMSO),8.6(s,1H),8.1(s,1H),7.7-7.9(m,2H),7.2-7.5(m,5H),5.6(d,1H),5.0(s,2H),3.8-4.1 (m,5H),3.0-3.8(m,5H),2.0-2.4(m,5H),1.0-1.8(m,3H).MS(ESI)m/z:507.2(M+H)+.

(4) NHWD-830 (Compound 9)

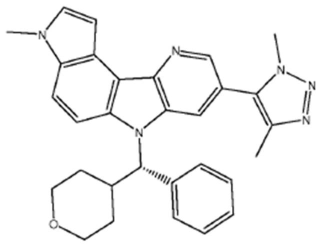

Compound 9

Step A

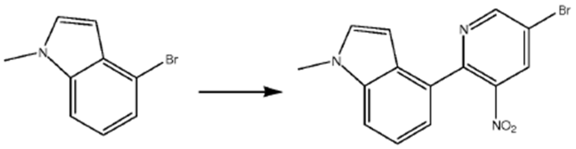

Compound 9a Compound 9b

To a mixed solution of compound 9a (5g, 24mmol), bisboronic acid pinacol (7.6g, 30mmol) and KOAc (5g,48mmol) in 1,4-dioxane (150ml) was added Pd(dppf)$_2$Cl$_2$ (1g), and flushed with nitrogen. The reaction flask was sealed and the reaction was stirred overnight at 85°C. After cooling to room temperature, an aqueous Na_2CO_3 solution (2.5M, 20ml), Pd(dppf)$_2$Cl$_2$ (1g) and 2,5-dibromo-3-nitropyridine (8g, 30mmol) were added. After flushed with nitrogen for 10 minutes, the reaction flask was sealed and the mixture was stirred overnight at 85°C. The reaction was poured into water and extracted with ethyl acetate. The mixed organic phase was dried over Na_2SO_4, dried by suction and purified by silica gel column chromatography (PE:EA=10:1 to 1:1) to provide compound 9b as a yellow solid.

HNMR(DMSO),9.0(s,1H),8.8(s,1H),7.5(m,1H),7.4(m,1H),7.2-7.3(m,2H),7.1-7.2(m,1H),3.8(s, 3H).MS(ESI)m/z:332.4(M+H)+.

Step B

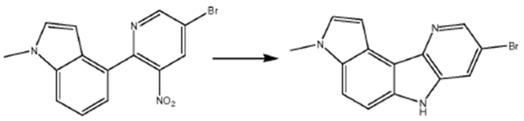

Compound 9b Compound 9c

Compound 9b (5g, 15mmol) was refluxed in triethylphosphite (50ml) solution for 1h. After the solvent was drained, water was added. The solid obtained was washed and dried to provide a compound 9c.

HNMR(DMSO),11.5(s,1H),8.5(s,1H),8.1(s,1H),7.7(m,1H),7.47(s,1H),7.4(m,1H),7.0(s,1H),3.9 (s,3H).MS(ESI)m/z:301.2 (M+H)+.

Step C

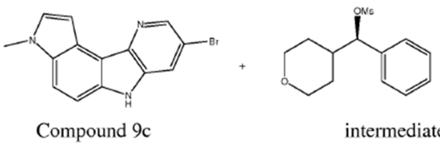

Compound 9c intermediate c

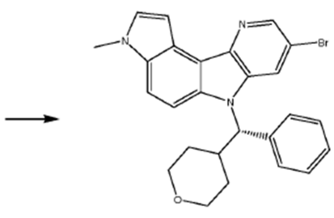

Compound 9d

Compound 9c (2 g, 6.6 mmol) was dissolved in dry DMF (30 ml), and Cs_2CO_3 (5 g, 14.4 mmol) and intermediate c (3.2 g, 13.2 mmol) were added. The reaction mixture was stirred at 45°C for three days and then intermediate c (3.2 g, 13.2 mmol) was added dropwise, and stirred at 50° C for three days. After the solution was drained, the crude product was purified by column chromatography on silica gel (DCM:MeOH=50:1 to 10:1) to obtain yellow compound 9d.

HNMR(CD3Cl),8.55(s,1H),7.96(s,1H),7.2-7.6(m,9H),5.4(d,1H),3.7-4.0(m,5H),3.0-3.4(m,3H), 1.0-2.0(m,4H).MS(ESI)m/z:474.2.0(M+H)+.

Step D

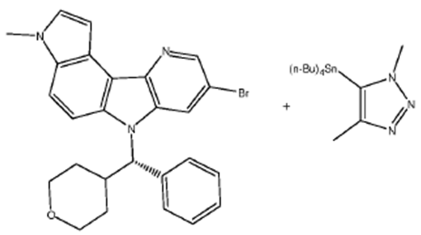

Compound 9d intermediate b

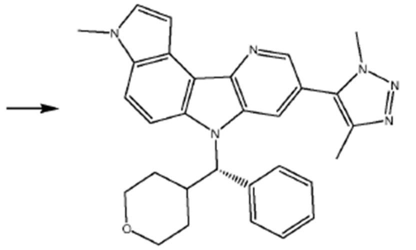

Compound 9

A mixed solution of compound 9d (0.8g, 1.7mmol), intermediate b (1.3g, 3.4mmol), triethylamine (0.6ml, 4.2mmol), and $Pd(dppf)_2Cl_2$ (200mg) in DMF (10ml) was flushed with nitrogen. The reaction flask was sealed, the mixture was heated and stirred at 100-140° C for 4h, poured into water, and extracted with DCM. The combined organic phases were dried and aspirated to dryness to give crude product purified by silica gel column chromatography (DCM:MeOH=50:1 to 10:1) to provide crude product as a yellow solid and purified by HPLC to obtain compound 9.

HNMR(CDCl3),8.88(s,1H),8.59(s,1H),7.8-7.9(m,2H),7.7(s,1H),7.2-7.6(m,6H),5.6(d,1H),4.1(m,1H),3.9(s,3H),3.8(m,1H),3.5(m,1H),3.3(m,1H),3.1(m,1H),2.3(s,3H),2.0(m,4H),1.4(m,1H),1.2(m,1H),1.05(m,1H).MS(ESI)m/z:491.8(M+H)+.

(5) NHWD-840 (Compound 4)

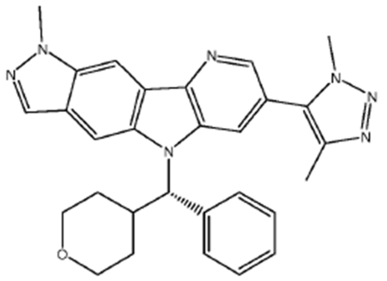

Compound 4

Step A

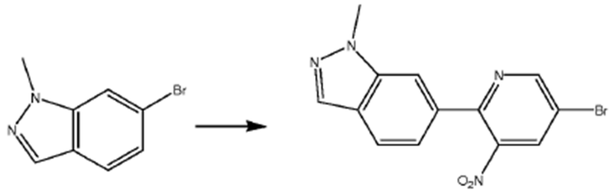

Compound 4a Compound 4b

To a mixed solution of compound 4a (10.5g, 50mmol), bisboronic acid pinacol (19g, 75mmol) and KOAc (7.5g, 75mmol) in 1,4-dioxane (150ml) was added $Pd(dppf)_2Cl_2$ (1g), and flushed with nitrogen. The reaction flask was sealed and the reaction was stirred overnight at 85°C. After cooling to room temperature, an aqueous Na_2CO_3 solution (2.5M, 30ml), $Pd(dppf)_2Cl_2$ (1g) and 2,5-dibromo-3-nitropyridine (21g, 75mmol) were added. After flushed with nitrogen for 10 minutes, the reaction flask was sealed and the mixture was stirred overnight at 85°C. The reaction was poured into water and extracted with ethyl acetate. The organic phase mixture was dried over Na_2SO_4, dried by suction and purified by silica gel column chromatography (PE:EA=10:1 to 1:1) to provide compound 4b as a yellow solid.

HNMR(CDCl3),8.9(s,1H),8.3(s,1H),8.0(s,1H),7.8(m,1H),7.7(m,1H),7.2(m,1H),4.1(s,3H).MS(ESI)m/z:332.8(M+H)+.

Step B

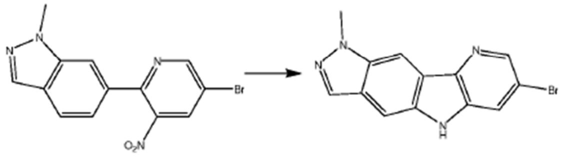

Compound 4b Compound 4c

Compound 4b (6.6g, 20mmol) was refluxed in triethylphosphite (80ml) solution for 3h. After the solvent was drained, water was added. The solid obtained was washed and dried to provide a compound 4c.

HNMR(CDCl3),8.5(s,1H),8.0-8.1(m,3H),7.5(s,1H),3.0(s,3H).MS(ESI)m/z:301.0 (M+H)+.

Step C

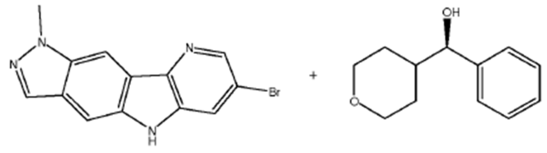

Compound 4c intermediate a

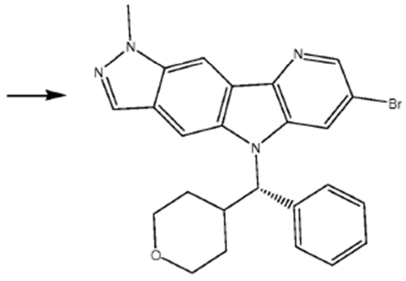

Compound 4d

Compound 4c (3g, 10mmol) was dissolved in DCM (250ml), and PPh3 (4g, 15mmol) and intermediate a (2.9g, 15mmol) were added. The reaction mixture was stirred in ice-water bath for 1 h, then a 30ml solution of DIAD (3g, 15mmol) in DCM was slowly added dropwise, and then stirred at room temperature, TLC monitored until the reaction is completed. After the solution was dried by suction, the crude was purified by silica gel column chromatography (DCM: MeOH = 50: 1 to 10: 1) to provide white compound 4d.

HNMR(CDCl3),8.6(s,1H),8.2(s,1H),8.0-8.1(m,1H),7.8(s,1H),7.6-7.7(m,2H),7.3-7.6(m,4H),6.0(d,1H),4.4(s,3H),3.9-4.0(m,1H),3.6-3.7(m,1H),3.4-3.5(m,1H),3.1-3.2(m,1H),2.8-3.0(m,1H),2.0-2.1(m,1H),0.8-1.4(m,2H),0.5(m,1H).MS(ESI)m/z:475.0(M+H)+.

Step D

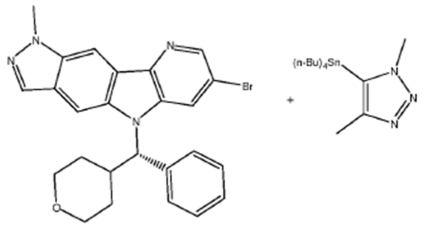

Compound 4d intermediate b

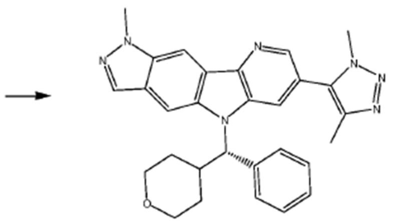

Compound 4

A mixed solution of compound 4d (1g, 2.1mmol), intermediate b (1.6g, 4.2mmol), triethylamine (0.6ml, 4.2mmol), $Pd(dppf)_2Cl_2$ (200mg) in DMF (20ml) was flushed with nitrogen. The reaction flask was sealed, the reaction was heated and stirred at 100-140° C for 4 h, poured into water, and extracted with DCM. The combined organic phases were dried and aspirated to dryness to give crude product purified by silica gel column chromatography (DCM:MeOH=50: 1 to 10:1) to provide crude product as a yellow solid purified by HPLC to obtain compound 4.

HNMR(CDCl3),8.54(s,1H),8.23(s,1H),8.17(d,1H),7.70(d,1H),7.5(m,3H),7.3-7.4(m,3H),6.22(d,1H),4.5(s,3H),4.0(m,1H),3.85(s,3H),3.6-3.7(m,1H),3.4-3.5(m,1H),3.0-3.1(m,1H),2.9-3.0(m,1H),2.26(s,3H),2.0-2.2(m,1H),1.0-1.4(m,2H),1.0(m,1H).MS(ESI)m/z:492.2(M+H)+.

(6) NHWD-850 (Compound 5)

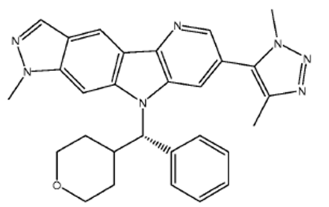

Compound 5

Step A

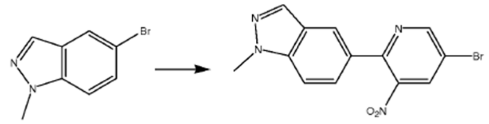

Compound 5a Compound 5b

To a mixed solution of compound 5a (10.5g, 50mmol), bisboronic acid pinacol (19g, 75mmol) and KOAc (7.5g, 75mmol) in 1,4-dioxane (150ml) was added $Pd(dppf)_2Cl_2$ (1g), and flushed with nitrogen. The reaction flask was sealed and the reaction was stirred overnight at 85°C. After cooling to room temperature, an aqueous Na_2CO_3 solution (2.5M, 30ml), $Pd(dppf)_2Cl_2$ (1g) and 2,5-dibromo-3-nitropyridine (21g, 75mmol) were added. After flushed with nitrogen for 10 minutes, the reaction flask was sealed and the mixture was stirred overnight at 85°C. The reaction was poured into water and extracted with ethyl acetate. The mixed organic phase was dried over Na_2SO_4, dried by suction and purified by silica gel column chromatography (PE:EA=10:1 to 1:1) to provide compound 5b as a yellow solid.

HNMR(CDCl3),8.9(d,1H),8.3(d,1H),8.0(s,1H),7.9(s,1H),7.5(m,1H),7.4(m,1H),4.1(s,3H).MS(ESI)m/z:332.8(M+H)+.

Step B

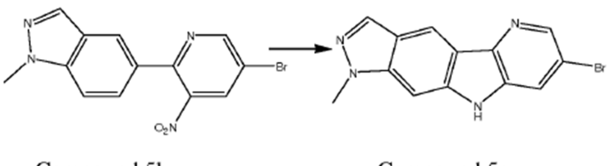

Compound 5b Compound 5c

Compound 5b (6.6g, 20mmol) was refluxed in triethylphosphite (80ml) solution for 3h. After the solvent was drained, water was added. The solid obtained was washed and dried to provide a compound 5c.

HNMR(CDCl3),8.5(s,1H),8.25(s,1H),8.18(s,1H),7.88(s,1H),7.2(s,1H),4.1(s,3H).MS(ESI)m/z: 301.0 (M+H)+.

Step C

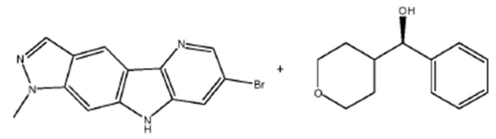

Compound 5c intermediate a

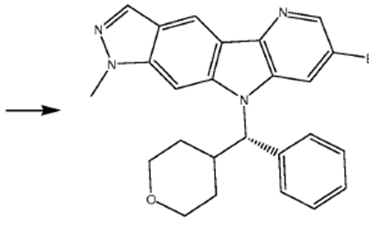

Compound 5d

Compound 5c (3g, 10mmol) was dissolved in DCM (200ml), and PPh3 (4g, 15mmol) and intermediate a (2.9g, 15mmol) were added. The reaction mixture was stirred in ice-water bath for 1 h, and then a 30ml solution of DIAD (3g, 15mmol) in DCM was slowly added dropwise, and then stirred at room temperature, TLC monitored until the reaction was completed. After the solution was dried by suction , the crude was purified by silica gel column chromatography (DCM: MeOH = 50: 1 to 10: 1) to provide white compound 5d. MS(ESI)m/z:475.0(M+H)+.

Step D

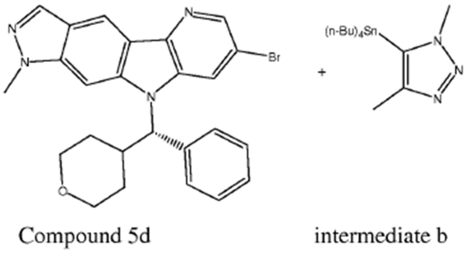

Compound 5d intermediate b

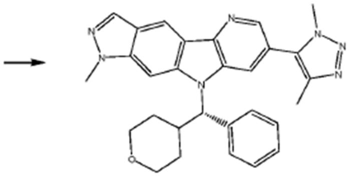

Compound 5

A mixed of compound 5d (1g, 2.1mmol), intermediate b (1.6g, 4.2mmol), triethylamine (0.6ml, 4.2mmol), Pd(dppf)$_2$Cl$_2$ (200mg) in DMF (20ml) was flushed with nitrogen. The reaction flask was sealed, the mixture was heated and stirred at 100-140° C for 4 h, poured into water, and extracted with DCM. The combined organic phases were dried and aspirated to dryness to give crude product purified by silica gel column chromatography (DCM:MeOH=50: 1 to 10:1) to provide crude product as a yellow solid purified by HPLC to obtain compound 5.

HNMR(DMSO),9.0(s,1H),8.52(s,1H),8.27(s,1H),8.2(d,1H),7.5-7.7(m,3H),7.2-7.4(m,3H),6.0(d,1H),4.3(s,3H),4.1(m,1H),3.95(s,3H),3.0-3.8(m,4H),2.26(s,3H),1.8-2.0(m,1H),1.0-1.4(m,3H).MS(ESI)m/z:492.2(M+H)+.

(7) NHWD-860 (Compound 7)

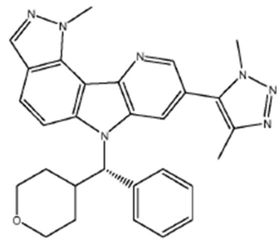

Compound 7

Step A

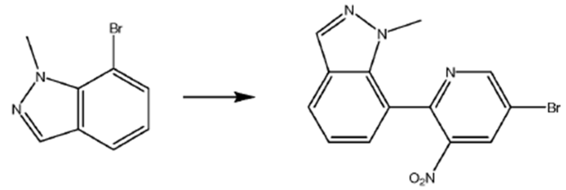

Compound 7a Compound 7b

To a mixed solution of compound 7a (10.5g, 50mmol), bisboronic acid pinacol (19g, 75mmol) and KOAc (7.5g, 75mmol) in 1,4-dioxane (150ml) was added Pd(dppf)$_2$Cl$_2$ (1g), and flushed with nitrogen. The reaction flask was sealed and the reaction was stirred overnight at 85°C. After cooling to room temperature, an aqueous Na_2CO_3 solution (2.5M, 30ml), Pd(dppf)$_2$Cl$_2$ (1g) and 2,5-dibromo-3-nitropyridine (21g, 75mmol) were added. After flushed with nitrogen for 10 minutes, the reaction flask was sealed and the mixture was stirred overnight at 85°C. The reaction was poured into water and extracted with ethyl acetate. The mixed organic phase was dried over Na_2SO_4, dried by suction and purified by silica gel column chromatography (PE:EA=10:1 to 1:1) to provide compound 7b as a yellow solid.

HNMR(DMSO),9.14(s,1H),8.8(s,1H),8.4(s,1H),7.85(m,1H),7.60(m,1H),7.2(m,1H),4.1(s,3H). MS(ESI)m/z:332.8(M+H)+.

Step B

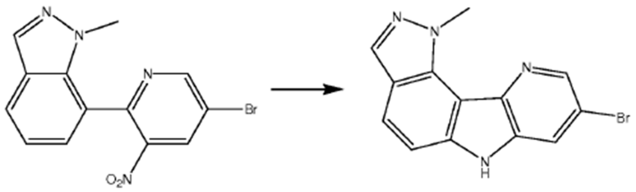

Compound 7b Compound 7c

Compound 7b (6.6g, 20mmol) was refluxed in triethylphosphite (80ml) solution for 3h. After the solvent was drained, water was added. The solid obtained was washed and dried to provide a compound 7c.

HNMR(DMSO),11.8(s,1H),8.55(s,1H),8.41(s,1H),8.14(s,1H),7.8(m,1H),7.35(m,1H),4.2(s,3H) .MS(ESI)m/z:301.0 (M+H)+.

Step C

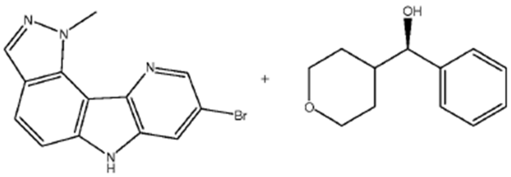

Compound 7c intermediate a

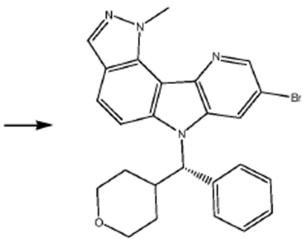

Compound 7d

Compound 7c (3g, 10mmol) was dissolved in DCM (200ml), and PPh3 (4g, 15mmol), intermediate a (2.9g, 15mmol) were added. The reaction mixture was stirred in ice-water bath for 1 h, and then 30ml of DIAD (3g, 15mmol) in DCM was slowly added dropwise, and then stirred at room temperature, TLC monitored until the reaction is completed. After the solution was dried by suction, the crude was purified by silica gel column chromatography (DCM: MeOH = 50: 1 to 10: 1) to provide white compound 7d.

HNMR(CDCl3),8.6(s,1H),8.1(s,1H),7.8(m,1H),7.4-7.5(m,3H),7.2-7.4(m,4H),5.5(d,1H),4.9(s,3 H),4.0-4.1(m,1H),3.8(m,1H),3.5(m,1H),3.3(m,1H),3.1(m,1H),2.0(m,1H),1.8(m,1H),1.2-1.4(m,1H), 0.8-1.0(m,1).MS(ESI)m/z:475.0(M+H)+.

Step D

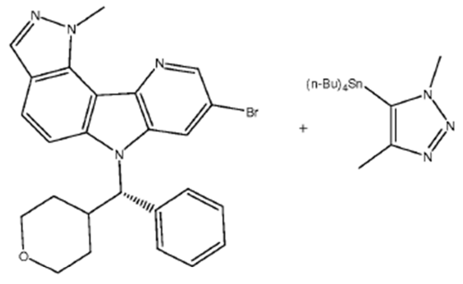

Compound 7d intermediate b

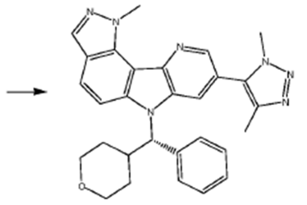

Compound 7

A mixed solution of compound 7d (1g, 2.1mmol), intermediate b (1.6g, 4.2mmol), triethylamine (0.6ml, 4.2mmol), and Pd(dppf)$_2$Cl$_2$ (200mg) in DMF (20ml) was flushed with nitrogen. The reaction flask was sealed; the mixture was heated and stirred at 100-140° C for 4 h, poured into water, and extracted with DCM. The combined organic phases were dried and aspirated to dryness to give crude product purified by silica gel column chromatography (DCM:MeOH=50: 1 to 10:1) to provide crude product as a yellow solid and purified by HPLC to obtain compound 7.

HNMR(DMSO),8.62(s,1H),8.15(s,1H),7.89(m,2H),7.68(m,2H),7.2-7.4(m,4H),5.93(d,1H),4.9(s,3H),4.0(s,3H),3.0-4.0(m,5H),2.31(s,3H),1.0-2.0(m,4H).MS(ESI)m/z:492.2(M+H)+.

(8) **NHWD-870 (Compound 8, see also Supplementary Figure 2a)**

To a 1 L round bottom flask containing compound 8a (10.5 g,50 mmol) in THF (150mL) was added bis(pincolato)diboron (19 g, 75mmol), potassium acetate (7.5 g,75 mmol) and followed by pd(dppf)2cl2 (1 g). N2 was bubbled into the mixture for 5 min and the resulting reaction mixture was heated at 80°C overnight. After cooling to RT, aqueous Na2CO3 (2.5 M, 30 mL) was added, followed by pd(dppf)2cl2 (1 g), and 2,5-dibromo-3-nitropyridine (21 g, 75 mmol). N2 was bubbled for 5 min and the resulting reaction mixture was heated at 80°C overnight, concentrated and then diluted with 10%NaCl

solution and extracted with EtOAc. The organic layer was concentrated and purified in silica gel chromatography (gradient from 10% to 50% EtOAc/PE) to get a slightly yellow solid 8b.

HNMR(CDCl3),8.99(s,1H),8.39(s,1H),7.9(s,1H),7.52(m,1H),7.45(m,1H),7.2(m,1H),4.1(s,3H).MS(ESI)m/z:332.8(M+H)+.

To a 500 mL round bottom flask containing compound 8b (6.6 g, 20 mmol) was added triethyl phosphite (80 mL). The reaction mixture was heated to reflux for 3h, concentrated, and diluted with water to have precipitation. It was filtered and dried to have a solid 8c.
HNMR(DMSO),11.8(s,1H),8.6(s,1H),8.4(s,1H),8.2(s,1H),7.85(m,1H),7.7(m,1H),4.1(s,3H).MS(ESI)m/z:301.0 (M+H)+.

To a 1 L round bottom flask containing compound 8c (3 g, 10 mml) in CH2Cl2 (200 mL) was added PPh3 (4 g, 15 mmol), followed by (R)-phenyl(tetrahydro-2H-pyran-4-yl)methanol (2.9 g, 15 mmol) (J. Org. Chem. 2006, 71, 7035-7044) in ice bath. The reaction mixture was stirred for 1 h in ice bath and then CH2Cl2 (30 mL) solution containing diisopropyl azodiformate (3 g, 15 mmol) was added dropwise. The reaction mixture was stirred at RT until the reaction is complete in TLC, concentrated, and purified in silica gel chromatography (gradient from 2% to 10% MeOH/CH2Cl2) to get a white solid 8d. MS(ESI)m/z:475.0(M+H)+.

To a 100 mL round bottom flask containing compound 8d (1 g, 2.1 mmol) in DMF (20 mL) was added 1,4-dimethyl-5-tributylstannyl-1H-1,2,3-triazole (1.6 g, 4.2 mmol)(Seefeld, M.A., et al. WO2008098104) , pd(dppf)2Cl2 (200 mg), triethylamine (0.6 mL, 4.2 mmol). N2 was bubbled through the mixture and the resulting mixture was heated at 100°C for 4 hour, diluted with 10% NaCl solution and extracted with CH2Cl2. The organic layer was concentrated and purified in silica gel chromatography (gradient from 2% to 10% MeOH/CH2Cl2) to get a slightly yellow solid which was washed with diethyl ether to have a white solid compound 8.

HNMR(CDCl3),8.72(s,1H),8.58(s,1H),7.8(m,1H),7.6-7.7(m,2H),7.4(m,2H),7.2-7.3(m,3H),5.6(d,1H),4.2(s,3H),4.0(m,1H),3.9(s,3H),3.8(m,1H),3.5(m,1H),3.3(m,1H),3.1(m,1H),2.3(s,3H),2.0(m,1H),1.4(m,1H),1.2(m,1H),1.05(m,1H).MS(ESI)m/z:492.2(M+H)+.

2. **BRD4 binding assay.** The inhibitory effects of NHWD-530, NHWD-540, NHWD-560, NHWD-900, NHWD-910, NHWD-920 and NHWD-930 on BRD4 binding to acetylated histone H4 were measured with BRD4 (BD1+BD2) TR-FRET Assay Kit (BPS, Cat # 32612) per manufacturer's instructions. The inhibitor concentrations used were 100, 1000 nM or 0.1, 0.2, 1, 4, 15.6, 62.5, 250, and 1,000 nM. DMSO was used as the control.

3. **AlamarBlue assay.** AlamarBlue was made by resazurin sodium salt (Sigma, Cat # 199303) dissolved in PBS. BMS-986158 and JQ1 were used as the controls. NCI-H211, MDA-MB231 and A375 cells were plated in 96-well plates at approximately 30% confluence and left overnight to adhere. In the following day, cells were treated with 0.1, 1, 10, 100 and 1,000 nM of NHWD-840, NHWD-850, NHWD-860, NHWD-870, JQ1, BMS-986158 or vehicle (0.1% DMSO). After 72 h of treatment, 10 μl AlamarBlue reagent (0.3 mg/ml) was added to each well, and after 18h, the florescence intensity in each well was measured using a top-reading fluorescent plate reader with excitation at 530 nm and emission at 590 nm.
4. **Protein extraction and western blot analysis.** Freshly dissected unfixed tissue was homogenized in lysis buffer. The lysates were centrifuged at 13,000g for 10 minutes at 4°C. Supernatants were collected and determined with a Bradford Protein Assay kit (Bio-Rad, Hercules, CA). The cell lysates were subjected to SDS-PAGE followed by immunoblotting (Immobilon P; Millipore, Milford, MA) with specific antibodies followed by detection using an enhanced chemiluminescence kit (Amersham Life Science, Arlington Heights, IL).
5. **Colony formation assay.** Cells were seeded in 6-well plates at 3,000 cells/well and treated with JQ1, NHWD-870, or DMSO control. Media were replaced once every week. After 2-3 weeks, depending on the cell growth rate, cells were fixed in 4% para-formaldehyde for 10 minutes at room temperature and stained with 0.5% crystal violet for 30 minutes. The plates were washed with water and dried before photographing. Colony numbers were calculated with imageJ version 1.52s [1].

6. **MTT and MTS assay.** Cells (5,000 cells per well) were seeded into 96-well plates. 20 μl MTT or MTS reagent was added into each well and incubated for 4 hours at 37℃, 5% CO_2. The optical density (OD490nm) was measured using a microplate reader, and the relative absorbance was calculated by subtracting the average background signal.

7. **Metabolic stability assay.** The metabolic profile of NHWD-870 was determined using mouse, rat, dog, monkey, and human liver microsomes using the protocols described below. Testosterone, Diclofenac, and Propafenone were included as the controls.

Materials and methods

No.	Compound ID	Exat Mass	Stock Conc. (mM)
Exp 1	NHWD-870	491.58	10
Control 1	Testosterone	288.42	10
Control 2	Diclofenac	295.14	10
Control 3	Propafenone	341.44	10

(1) Buffers:

1) 100 mM potassium phosphate buffer, PH7.4; 2) 10mM $MgCl_2$

(2) Compound Dilution:

1) Intermediate solution: Dilute 5 μL of compound or control from stock solution (10 mM) with 45 uL DMSO,then mixed with 450 μL 1:1 Methanol/Water (Conc.: 100 μM, 45%MeOH)
2) Working solution: Dilute 50 μL from intermediate solution with 450 μL 100 mM potassium phosphate buffer (Conc.: 10 μM, 4.5%MeOH)

(3) NADPH regenerating system (final Isocitric dehydrogenase conc.= 1 unit/mL at incubation):

1) β-Nicotinamide adenine dinucleotide phosphate, Vendor: sigma Cat. No. N0505
2) Isocitric acid, Vendor: Sigma Cat. No. I1252
3) Isocitric dehydrogenase, Vendor: sigma Cat. No. I2002

(4) Liver microsome solution preparation (final concentration of 0.5 mg protein/mL)

Microsomes	Product	Source
Human liver microsomes	Cat No.452117	BD Gentest
Rat liver microsome	Cat No. R1000	Xenotech
Mouse liver microsome	Cat No.M1000	Xenotech
Beagle dog liver microsome	Cat No.C1000	Xenotech
Rhesus monkey microsome	Cat No.P1000	Xenotech

(5) Stop solution:

Cold ACN including 100 ng/mL Tolbutamide and 100 ng/mL Labetalol as internal

standard (IS)

(6) Procedures

1) Add 10 μL compound or control working solution/well to all plates (T0, T5, T10, T20, T30, T60, NCF60) except matrix blank.

2) Dispense 680 μL/well microsome solution to 96-well plate as reservoir according to the

plate map. Then add 80 μL/well to every plate by Apricot, incubate the mixture of microsome solution and compound at 37℃ for about 10 min.

3) Add 10 μL 100 mM potassium phosphate buffer/well to NCF60, incubate at 37℃, start timer 1.

4) After pre-warming, dispense 90 μL/well NADPH regenerating system to 96-well plate as reservoir according to the plate map. Then add 10 μL/well to every plate by Apricot to start reaction.

5) Incubate at 37℃, start timer 2.

6) Add 300 (μL/well) stop solution (cold in 4℃, including 100 ng/mL Tolbutamide and 100 ng/mL Labetalol) to terminate the reaction.

7) The sampling plates are shaked for approx 10 min.

8) Samples are centrifuged at 4000 rpm for 20 min under 4℃.

9) While centrifuging, load 8×new 96-well plate with 300 μL HPLC water, then transfer 100 μL supernatant, mixed for LC/MS/MS.

(7) Data Analysis

Use equation of first order kinetics to calculate t1/2 and Clint(mic):

Equation of first order kinetics:

$$C_t = C_0 \bullet e^{-k_e \bullet t}$$

$$when\ C_t = \frac{1}{2} C_0 \ ,$$

$$T_{1/2} = \frac{Ln2}{k_e} = \frac{0.693}{k_e}$$

$$CL_{\text{int}(mic)} = \frac{0.693}{In\ vitro\ T_{1/2}} \bullet \frac{1}{mg / mL\ microsomal\ protein\ in\ reaction\ system}$$

$$CL_{\text{int}(liver)} = CL_{\text{int}(mic)} \bullet \frac{mg\ microsomes}{g\ liver} \bullet \frac{g\ liver}{kg\ body\ weight}$$

8. Equilibrium dialysis assay. Binding with plasma protein and recovery rate of NHWD-870 were performed with human, CD-1 mouse and SD rat plasma as described [2]. Warfarin was used as the positive control. The concentration was calculated using the peak area ratio of analyte and internal standard. The results were calculated by the following equations: % Unbound = 100 *FC / TC, % Bound = 100 * (1-FC / TC); % Recovery = 100 * (FC + TC) / T0; TC = Total compound concentration as determined by the calculated concentration on the retentate side of the membrane; FC = Free compound concentration as determined by the

calculated concentration on the dialysate side of the membrane; T0 = Total compound concentration as determined before dialysis. Good plasma stability should have a value of not less than 80% bound, and the recovery rate should be at least 30%.

9. **In vivo pharmacokinetics study.** Mouse and SD rat models were used to evaluate pharmacokinetic property of NHWD-870. NHWD-870 was administrated by either intravenous injection (IV, 1.75 mg/kg for mouse and 1 mg/kg for rat) or oral gavage (PO, 5 mg/kg) into mice or rats. Concentration of drugs in plasma, lung and tumor tissues were measured for the indicated time.
10. **hERG channel assay**. The potential inhibitory effect of NHWD-870 on human Ether-à-go-go related gene (hERG) channel was evaluated by manual patch-clamp system using the protocols described below. HEK293 cell line stably transfected with hERG gene was used in this study and Dofetilide was used as the benchmark for the quality of the assay. The potency of a test compound inhibiting hERG channel is list as follows: a) Low: IC_{50} > 10 μM; b) Moderate: 1 μM < IC_{50} < 10 μM; c) High: IC_{50} < 1 μM. The FDA criterion for defining a drug as hERG-positive is when IC_{50} < 1 μM.

hERG channel assay protocol

(1) Materials and Instrumentation

Materials and Instrumentation	Vendor (Cat #)
G418	Invitrogen (Cat. 11811031)
Blasticidin	Invitrogen (Cat. R21001)
Doxycycline	Sigma (Cat. D9891)
Multiclamp 700B Amplifier	AXON
EPC10 Amplifier	HEKA
Microscope	Olympus TH4-200
Perfusion system	ALA VM8 gravity-flow delivery system

(2) Cell lines and cell culture

hERG stably expressed HEK 293 cell line (Cat# K1236) was purchased from Invitrogen. The cells are cultured in 90% DMEM, 10% dialyzed FBS, 0.1 mM NEAA, 25 mM HEPES, 100 U/mL Penicillin-Streptomycin and 5 μg/mL Blasticidin and 400 μg/mL Geneticin. Cells are split using TrypLE™ Express about three times a week, and maintained between ~40% to ~80% confluence. Before the assay, the cells were onto the coverslips at 5 × 105 cells /per 6 cm cell culture dish and induced with doxycycline at 1 μg/mL for 48 hours.

(3) Solution preparations

1) Extracellular solution (in mM): 132 NaCl, 4 KCl, 3 CaCl2, 0.5 MgCl2, 11.1 glucose, and 10 HEPES (pH adjusted to 7.35 with NaOH);

2) Intercellular solution (in mM): 70 KF, 60 KCl, 15 NaCl, 5 EGTA, and 5 HEPES (pH adjusted to 7.35 with KOH).

(4) Working solution preparation for test compound

1) Test compounds were initially prepared in DMSO with final concentration of 30 mM as stock solution, respectively according to SOP-ADMET-MAN-007.

2) Then stock solution of each compound was serial-diluted by ratio of 1:3 with DMSO to prepare additional 4 intermedial solutions, which are10, 3.33, 1.11, 0.37 mM, respectively.

3) Right before the experiment, the working solutions are finally prepared by dilution of above described intermedial solutions in 1000 folds using extracellular solution. The final DMSO concentration was 0.1% except for 30 μM working solution in which DMSO was 0.3%.

4) hERG current in presence of 5 doses were tested for IC50 determination, consisting of 30, 10, 3.33, 1.11 and 0.37 μM.

(5) Experimental procedure

1) Remove the coverslip from the cell culture dish and place it on the microscope stage in bath chamber.

2) Locate a desirable cell using the ×10 objective. Locate the tip of the electrode under the microscope using the ×10 objective by focusing above the plane of the cells. Once the tip is in focus, advance the electrode downwards towards the cell using the coarse controls of the manipulator, while simultaneously moving the objective to keep the tip in focus.

3) When directly over the cell, switch to the ×40 objective and use the fine controls of the manipulator to approach the surface of the cell in small steps.

4) Apply gentle suction through the side-port of the electrode holder to form a gigaohm seal.

5) Use the Cfast to remove the capacity current that is in coincidence with the voltage step. Obtain the whole cell configuration by applying repetitive, brief, strong suction until the membrane patch has ruptured.

6) Set membrane potential to -60 mV at this point to ensure that hERG channels are not open. The spikes of capacity current should then be cancelled using the C_{slow} on the amplifier.

7) Set holding potential to -90 mV for 1 second; record current at 50 kHz and filter at 10 kHz. Leaking current was tested at -80 mV for 500 ms.

8) The hERG current was elicited by depolarizing at +30 mV for 4.8seconds and then the voltage was taken back to -50 mV for 5.2 seconds to remove the inactivation and observe the deactivating tail current. The maximum amount of tail current size was used to determine hERG current amplitude.

9) Record current for 120 seconds to assess the current stability. Only stable cells with recording parameters above threshold were applied for the drug administrations.

10) Firstly vehicle control was applied to the cells to establish the baseline. Once the hERG current was found to be stabilized for 3 minutes, compound was applied. hERG current

in the presence of test compound were recorded for approximately 5 minutes to reach steady state and then 5 sweeps were captured. For dose response testing, 5 doses of compound was applied to the cells accumulatively from low to high concentrations. In order to ensure the good performance of cultured cells and operations, the positive control, Dofetilide, with 5 dosing concentration was also used test the same batch of cells that were used for compounds.

(6) Data analysis

(6.1) Data acceptance criteria

The following criteria were used to determine data acceptability.

1) Initial seal resistance > 1 GΩ;

2) Leak currents < 50% of the control peak tail currents at any time;

3) The peak tail amplitude >250pA;

4) Membrane resistance Rm > 200 MΩ;

5) Access resistance (Ra) < 15 MΩ;

6) Apparent run-down of peak current < 2.5% per min.

(6.2) Data analysis

Data that met the above criteria for hERG current quality were further analyzed as the following steps.

1) Percent current inhibition was calculated using the following equation.

Note: PatchMaster software was used to extract the peak current from the original data.

$$\text{Peak current inhibition} = \left(1 - \frac{\text{Peak tail current}_{\text{compound}}}{\text{Peak tail current}_{\text{vehicle}}}\right) \times 100$$

2) The dose response curve of test compounds was plotted with %inhibition against the concentration of test compounds using Graphpad Prism 5.0, and fit the data to a sigmoid dose-response curve with a variable slope.

11. Plasmids and CRISPR/Cas9-mediated knockout. pCAG-HIF1α plasmid was purchased from Addgene (#21101) and was described previously [3]. sgRNAs were designed using CHOPCHOP (https://chopchop.rc.fas.harvard.edu/) and cloned into LentiCRISPRv2. Knockout cells were generated as described previously [4]. Briefly, 293T cells in 6-well plates were introduced with 1.5 μg LentiCRISPRv2 sgRNA plasmid, 1 μg psPAX2, and 0.5 μg pMD2.G. At 48 hours after transfection, lentivirus-containing media were collected and filtered through a 0.45 μm filter before being used to infect cells. Cells were infected with lentiviruses for 24 hours, then refed with fresh medium with puromycin. sgRNA controls were described previously[5]. sgRNA targeting sequences for BRD4 are: #1 AGACCAACCAACTGCAATACCT, #2 GAGTCTGGGATGTTCGTCTCTC.

12. **Luciferase reporter assays.** HeLa cells were seeded into 24-well plates, transfected with the control reporter pSV-*Renilla* and HIF1 firefly luciferase reporter p2.1, constructed with a fragment of the *ENO1* promoter [6], and exposed to 20% or 1% O_2 for 24 hours. Luciferase activities were measured using the Dual Luciferase Reporter Assay System (Promega). HIF1 reporter activity was determined by dividing the activity of firefly luciferase reporter by that of the Renilla luciferase control.

13. **Patients and tissue samples.** Following Institutional Review Board approval, a total of 128 patients with advanced epithelial ovarian cancer (EOC) from the research files at The Tumor Hospital of Harbin Medical University who were seen from January 2005 to December 2009, and who met our inclusion criteria, were included in this study. The eligibility criteria included the following: (1) pathologic examination confirming the presence of stage III EOC; (2) complete basic clinical data; (3) absence of any prior treatment for cancer; (4) no serious complications or other malignant disease; (5) the patients and family members being informed about the illness and having given informed consent before treatment. All patients had undergone complete cytoreductive surgery.

14. **Immunohistochemical staining.** Paraffin-embedded implantation samples isolated from peritoneal metastasis of EOC patients (128 cases) were sectioned at a thickness of 4μm. To stain CD68, CSF1 and p-BRD4, the slides were first deparaffinized in xylene and rehydrated with gradient concentrations of alcohol under standard procedures. After rehydration, the slides were immersed in 0.01 mol/L citrate buffer (pH 6.0) and heated (95°C) for 15 min for antigen retrieval. Then, the samples were incubated with 3% hydrogen peroxide (H_2O_2) for 10 minutes followed by 10% normal goat serum blocking for 10 minutes. Subsequently, the sections were incubated with rabbit polyclonal anti-human CD68 antibody (dilution 1:100) (sc-20060, Santa Cruz Biotechnology) and anti-human p-BRD4

(dilution 1:100), or with mouse monoclonal anti-human CSF1 (dilution 1:100) (sc-365779 Santa Cruz Biotechnology) for 1 hour at room temperature. After washing with PBST for 3 times, the sections were incubated with biotin-labeled secondary antibody followed by horseradish peroxidase (HRP)-conjugated streptavidin for 30 minutes individually at room temperature. After applying HRP substrate, 3.3'-diaminobenzidine tetrahydrochloride (D3939-1set, Sigma) in 0.01% H_2O_2, for 10 minutes, the slides were counterstained with Meyer's hematoxylin for 30 to 60 seconds and mounted with mounting medium for visualization under microscope [7,8]. Scoring of CD68, CSF1 and p-BRD4 in EOC samples via IHC staining follows the methods previously published [7,8]. Briefly, CD68, p-BRD4 and CSF1 staining intensity was determined using ImageJ version 1.52s [1], and normalized with cell area. Cell area was determined by manual delineation of raw IHC staining images. A minimum of 12 cells were analyzed from two independent experiments. All IHC staining samples from EOC patients were evaluated independently by two experienced pathologists.

15. ChIP-qPCR analysis.

A2780 cells were treated with 15nM NHWD-870 or DMSO for 3 days followed by cross-linking in DMEM with 1% formaldehyde for 10 minutes. After stopping the cross-linking by 0.125 M glycine, the cells were washed with cold PBS. Then the cells were scraped into Lysis Buffer 1 [50 mM Hepes-KOH (pH 7.5), 140 mM NaCl, 1 mM EDTA (pH 8.0), 10% Glycerol, 0.5% NP-40, 0.25% Triton X-100] and shook for 10 minutes at 4°C. Next the cells were pelleted and resuspended in Lysis Buffer 2 [(200 mM NaCl,1mM EDTA (pH 8.0), 0.5 mM EGTA, 10mM Tris (pH 8.0)) followed by 10-minute shaking at room temperature. Again the samples were pelleted and then resuspended in Lysis Buffer 3 [(1mM EDTA (pH 8.0), 0.5 mM EGTA, 10mM Tris (pH 8.0)). Next the chromatins were sonicated for 4 minutes using Bioruptor® Plus, diagenode at high energy followed by incubating with BRD4 (E2A7X) Rabbit mAb (Cell Signaling Technology #13440) or rabbit IgG (Beyotime A7016) and Protein A+G Agarose (Fast Flow, for IP) (Beyotime P2055) for immunoprecipitation over night at 4°C. The next day the samples were

washed for 5 times and then vortexed for 20 minutes at 65°C with TE buffer with 1% SDS for DNA extraction, followed by incubation at 65°C overnight for reverse cross-linking. The next day RNase A and proteinase K were added and incubated at 37°C for 2 hours, respectively. Then SanPrep Column PCR Product Purification Kit (Sangon Biotech) was used for DNA purification. ChIP-qPCR analyses were performed using primers F-"CGCGAACGACAAGAAAAAGT", R-"GAGCAGCAGCAGAAACAAAA" for the *HIF1A* promoter.

Supplementary References

1. Burgess A et al. Loss of human Greatwall results in G2 arrest and multiple mitotic defects due to deregulation of the cyclin B-Cdc2/PP2A balance. *Proc Natl Acad Sci U S A* **107**, 12564-9 (2010).

2. Curran RE, Claxton CR, Hutchison L, Harradine PJ, Martin IJ, Littlewood P. Control and measurement of plasma pH in equilibrium dialysis: influence on drug plasma protein binding. *Drug Metab Dispos* **39**, 551-557 (2011).

3. Chen B, Cepko CL. HDAC4 regulates neuronal survival in normal and diseased retinas. *Science* **323**, 256-259 (2009).

4. Cao J*, et al.* An easy and efficient inducible CRISPR/Cas9 platform with improved specificity for multiple gene targeting. *Nucleic Acids Res* **44**, e149 (2016).

5. Gale M*, et al.* Screen-identified selective inhibitor of lysine demethylase 5A blocks cancer cell growth and drug resistance. *Oncotarget* **7**, 39931-39944 (2016).

6. Luo W, Chang R, Zhong J, Pandey A, Semenza GL. Histone demethylase JMJD2C is a coactivator for hypoxia-inducible factor 1 that is required for breast cancer progression. *Proc Natl Acad Sci U S A* **109**, E3367-3376 (2012).

7. Yin M*, et al.* Tumor-associated macrophages drive spheroid formation during early transcoelomic metastasis of ovarian cancer. *J Clin Invest* **126**, 4157-4173 (2016).

8. Yin M, et al. ASK1-dependent endothelial cell activation is critical in ovarian cancer growth and metastasis. *JCI Insight* **2**, (2017).
