# Supplementary material for: Potent BRD4 inhibitor suppresses cancer cell-macrophage interaction
Source: Nat Commun. 2020 Apr 14;11:1833. doi: 10.1038/s41467-020-15290-0 (PMC7156724; doi:10.1038/s41467-020-15290-0)
Supplement: Supplementary file 3 — Reporting Summary [file 41467_2020_15290_MOESM3_ESM.pdf]

nature research

Corresponding author(s): Qin Yan, Mingzhu Yin, Xiang Chen

Last updated by author(s): Feb 10, 2020

# Reporting Summary

Nature Research wishes to improve the reproducibility of the work that we publish. This form provides structure for consistency and transparency in reporting. For further information on Nature Research policies, see Authors & Referees and the Editorial Policy Checklist .

## Statistics

For all statistical analyses, confirm that the following items are present in the figure legend, table legend, main text, or Methods section.

| n/a | Confirmed | |
|---|---|---|
| ☐ | ☒ | The exact sample size (*n*) for each experimental group/condition, given as a discrete number and unit of measurement |
| ☐ | ☒ | A statement on whether measurements were taken from distinct samples or whether the same sample was measured repeatedly |
| ☐ | ☒ | The statistical test(s) used AND whether they are one- or two-sided<br>*Only common tests should be described solely by name; describe more complex techniques in the Methods section.* |
| ☐ | ☒ | A description of all covariates tested |
| ☐ | ☒ | A description of any assumptions or corrections, such as tests of normality and adjustment for multiple comparisons |
| ☐ | ☒ | A full description of the statistical parameters including central tendency (e.g. means) or other basic estimates (e.g. regression coefficient) AND variation (e.g. standard deviation) or associated estimates of uncertainty (e.g. confidence intervals) |
| ☐ | ☒ | For null hypothesis testing, the test statistic (e.g. *F*, *t*, *r*) with confidence intervals, effect sizes, degrees of freedom and *P* value noted<br>*Give P values as exact values whenever suitable.* |
| ☒ | ☐ | For Bayesian analysis, information on the choice of priors and Markov chain Monte Carlo settings |
| ☒ | ☐ | For hierarchical and complex designs, identification of the appropriate level for tests and full reporting of outcomes |
| ☒ | ☐ | Estimates of effect sizes (e.g. Cohen's *d*, Pearson's *r*), indicating how they were calculated |

*Our web collection on statistics for biologists contains articles on many of the points above.*

## Software and code

Policy information about availability of computer code

| | |
|---|---|
| Data collection | FACS data: FACSCalibur (BD Biosciences); RT-qPCR and ChIP-qPCR, Biorad CFX96 Real time system; Image data: Openlab3 |
| Data analysis | Statistical analysis: SAS version 9.1.4; RT-qPCR and ChIP-qPCR: CFX Manager version 3.1.1517.823, Microsoft Excel version 2010; FACS data: BD CellQuest Pro version 5.1; Image data: ImageJ version 1.52s |

For manuscripts utilizing custom algorithms or software that are central to the research but not yet described in published literature, software must be made available to editors/reviewers. We strongly encourage code deposition in a community repository (e.g. GitHub). See the Nature Research guidelines for submitting code & software for further information.

## Data

Policy information about availability of data

All manuscripts must include a data availability statement. This statement should provide the following information, where applicable:
- Accession codes, unique identifiers, or web links for publicly available datasets
- A list of figures that have associated raw data
- A description of any restrictions on data availability

The ChIP-seq data referenced in this study are available under GSE89129 and GSE102409 in Gene Expression Omnibus. All the data supporting this study are available within the article, the Supplementary file, the Source Data file, and from the corresponding authors upon reasonable request.

# Field-specific reporting

Please select the one below that is the best fit for your research. If you are not sure, read the appropriate sections before making your selection.

# Life sciences study design

All studies must disclose on these points even when the disclosure is negative.

| | |
|---|---|
| Sample size | Sample sizes were chosen based on power analysis of the phenotype assumpution and were sufficient for statistical analysis |
| Data exclusions | No data was excluded from the analysis |
| Replication | Most experiments were reproduced three times unless indicated in the figure legends. |
| Randomization | Animals used for treatment were randomized to each group. No randomization was applied to other experiments. |
| Blinding | Analysis of IHC staining of human samples were performed in a blinding manner by two pathologists as these experiments are more subject to human bias. Other experiments were not performed in a blinding manner. Quantifications with ImageJ were done in an unbiased way. |

# Reporting for specific materials, systems and methods

We require information from authors about some types of materials, experimental systems and methods used in many studies. Here, indicate whether each material, system or method listed is relevant to your study. If you are not sure if a list item applies to your research, read the appropriate section before selecting a response.

## Materials & experimental systems

| n/a | Involved in the study | |
|---|---|---|
| ☐ | ☒ | Antibodies |
| ☐ | ☒ | Eukaryotic cell lines |
| ☒ | ☐ | Palaeontology |
| ☐ | ☒ | Animals and other organisms |
| ☐ | ☒ | Human research participants |
| ☒ | ☐ | Clinical data |

## Methods

| n/a | Involved in the study | |
|---|---|---|
| ☒ | ☐ | ChIP-seq |
| ☐ | ☒ | Flow cytometry |
| ☒ | ☐ | MRI-based neuroimaging |

## Antibodies

| | |
|---|---|
| Antibodies used | List of antibodies in supplementary Table 8 and method. β-Actin Gene Tex Inc Rabbit GTX109639;AKT1/2/3 Santa Cruz Rabbit sc-8312; p-AKT1 Cell signaling Rabbit #4060; BRD4 abcam Rabbit ab128874; BRD4 Cell signaling Rabbit #13440; CD68 abcam Mouse ab31630; CD68(KP1) Santa Cruz Mouse sc-20060; Cleaved caspase 3 Cell signaling Rabbit #9661; c-MYC Santa Cruz Rabbit SC-40; E-Cadherin abcam Mouse ab15148; EGF abcam Rabbit ab9695; ERK Santa Cruz Rabbit sc-94; p-ERK Cell signaling Mouse #9106; GAPDH Cell signaling Rabbit #2118; Ki67 Cell signaling Rabbit #9129; CSF1 Santa Cruz Mouse sc-365779; CSF1 abcam Rabbit ab52864; CSF1R/CD115 R&D systems Inc Mouse MAB3291-100 (clone 61701); PI3K Santa Cruz Mouse sc-1637; p-PI3K Cell signaling Rabbit #4228; Mouse anti-Human IgG1 abcam Mouse ab1927; HIF1α Novusbio Rabbit Nb100-105; Rabbit IgG Beyotime Rabbit A7016; PE-F4/80 Biolegend Rat 123109; APC-F4/80 Biolegend Rat 100311; FITC-CD11b Biolegend Rat 101205; PE-CD206 Biolegend Rat 141705; FITC-Ki67 Ebioscience Rat 11-5698-80; APC-CD45 Biolegend Rat 103112, p-BRD4 rabbit polyclonal antibody was a gift from Dr. Cheng-Ming Chiang, University of Texas Southwestern Medical Center. |
| Validation | Appropriate controls were used to validate the antibodies. Commercially available antibodies were validated by the supplier and by us using appropriate controls. For example, BRD4 antibody was validated by western blot using BRD4 knockout cells. CSF1 antibody was validated by CSF1 overexpression. Please refer to the vendor's websites for further details. |

## Eukaryotic cell lines

Policy information about cell lines

| | |
|---|---|
| Cell line source(s) | Cell lines HEK293T, MCF-10A, SKBR3, UACC812, MAD-MB361, MDA-MB231, A2780, IGROV1, SKOV3, ECC-1, Ishikawa, HELA, A549, A375, ID8, B16, B16F10 , H1975, NCI-H211, JER, NCI-H526, NCI-H69, JEG-3, Hep G2, U251, SH-SY5Y, ES-2, HUVEC, SUM149 and SUM159 were acquired from the American Type Culture Collection (ATCC, Manassas, VA),or colleagues, and YUSOC, YUGASP, YUAME and YUMAC were acquired from the Specimen Resource Core of Yale SPORE in skin cancer. |
| Authentication | Cell lines were early passages from these resources and were not authenticated. |
| Mycoplasma contamination | Cell lines were routinely tested for be mycoplasma free. |
| Commonly misidentified lines (See ICLAC register) | No commonly misidentified cell lines were used. |

## Animals and other organisms

Policy information about studies involving animals; ARRIVE guidelines recommended for reporting animal research

| | |
|---|---|
| Laboratory animals | 4-6 weeks old female BALB/c nude mice were used for most experiments, with the exception of 6-8 weeks old female C57BL/6 mice were used for B16F10 experiments, and 6-8 weeks old male BALB/c nude mice and SD rats were used for pharmacokinetic experiments. All animals were purchased from Shanghai SIPPR-Bk Lab Animal Co., Ltd. All animals were housed under a regimen of 12 h light/12 h dark cycles and specific pathogen-free conditions. |
| Wild animals | No wild animals were involved in this study. |
| Field-collected samples | No field-collected samples were used in this study. |
| Ethics oversight | All the procedures were evaluated and approved by the Institutional Animal Care and Use Committee of Central South University and Yale University. |

Note that full information on the approval of the study protocol must also be provided in the manuscript.

## Human research participants

Policy information about studies involving human research participants

| | |
|---|---|
| Population characteristics | Patients with advanced epithelial ovarian cancer (EOC) from the research files at The Tumor Hospital of Harbin Medical University who were seen from January 2005 to December 2009, and who met our inclusion criteria, were included in this study. The eligibility criteria included the following: (1) pathologic examination confirming the presence of stage III EOC; (2) complete basic clinical data; (3) absence of any prior treatment for cancer; (4) no serious complications or other malignant disease; (5) the patients and family members being informed about the illness and having given informed consent before treatment. All patients had undergone complete cytoreductive surgery. |
| Recruitment | Patient materials used in this study were archived materials. The patients were recruited with informed consent for treatment and sample collection. |
| Ethics oversight | The study was approved by Institutional Review Board of Central South University and The Tumor Hospital of Harbin Medical University |

Note that full information on the approval of the study protocol must also be provided in the manuscript.

## Flow Cytometry

### Plots

Confirm that:

- [x] The axis labels state the marker and fluorochrome used (e.g. CD4-FITC).
- [x] The axis scales are clearly visible. Include numbers along axes only for bottom left plot of group (a 'group' is an analysis of identical markers).
- [x] All plots are contour plots with outliers or pseudocolor plots.
- [x] A numerical value for number of cells or percentage (with statistics) is provided.

### Methodology

| | |
|---|---|
| Sample preparation | Tumor tissues and tumor spheroids were collected from 3 ml ascitic fluid by using Falcon™ cell strainers in A2780 or A375 tumor models. |
| Instrument | Flow cytometry was performed on a FACSCalibur (BD Biosciences). |
| Software | Data were analyzed with BD CellQuest Pro software. |
| Cell population abundance | Cell population were quantified using FACS plots. |
| Gating strategy | Cells were first gated to exclude debris and non-singlets, the targeted cells were gated by cell surface markers using IgG as the negative control to make the cutoff. |

- [x] Tick this box to confirm that a figure exemplifying the gating strategy is provided in the Supplementary Information.
